# DNA barcoding facilitates associations and diagnoses for Trichoptera larvae of the Churchill (Manitoba, Canada) area

**DOI:** 10.1186/1472-6785-13-5

**Published:** 2013-02-20

**Authors:** David E Ruiter, Elizabeth E Boyle, Xin Zhou

**Affiliations:** 1 235 SW Central Avenue, Grants Pass, OR 97526, USA; 2 Department of Integrative Biology, University of Guelph, 50 Stone Rd. E, Guelph, ON N1G 2 W1, Canada; 3 BGI-Shenzhen, Beishan Rd., Yantian District, Shenzhen, Guangdong Province 518083, China

**Keywords:** Caddisfly, Freshwater, Life history, Ecology, Biomonitoring, DNA taxonomy, DNA barcoding, Barcoding biotas

## Abstract

**Background:**

The North American Trichoptera larvae are poorly known at the species level, despite their importance in the understanding of freshwater fauna and critical use in biomonitoring. This study focused on morphological diagnoses for larvae occurring in the Churchill, Manitoba area, representing the largest larval association effort for the caddisflies at any given locality thus far. The current DNA barcode reference library of Trichoptera (available on the Barcode of Life Data Systems) was utilized to provide larval-adult associations.

**Results:**

The present study collected an additional 23 new species records for the Churchill area, increasing the total Trichoptera richness to 91 species. We were able to associate 62 larval taxa, comprising 68.1% of the Churchill area Trichoptera taxa. This endeavor to identify immature life stage for the caddisflies enabled the development of morphological diagnoses, production of photographs and an appropriate taxonomic key to facilitate larval species analyses in the area.

**Conclusions:**

The use of DNA for associations of unknown larvae with known adults proved rapid and successful. This method should accelerate the state-of-knowledge for North American Trichoptera larvae as well as other taxonomic lineages. The morphological analysis should be useful for determination of material from the Churchill area.

## Background

Trichoptera (caddisflies) are a diverse group of insects composed of approximately 13,000 described species worldwide from 45 extant families [1]. This diversity, in part, is thought to be attributed to the ecological variation of their aquatic larvae. Trichoptera larvae display a wide-range of ecological diversity by exploiting a variety of habitat types and occupying various trophic levels by temporally differentiating as well as employing different life strategies [2]. This aquatic larval stage is also sensitive to environmental stressors, such as pesticides, nutrients, and sediments [3,4]. As such, Trichoptera larvae are commonly used for biomonitoring of freshwater habitats as general indicators of water quality and habitat.

However, the utility of these approaches has been impeded by the inability to correctly identify Trichoptera larvae with a high level of taxonomic resolution. This problem is due to the majority of the North American taxonomic literature for Trichoptera being written for adults, in addition to the technical challenges involved in conventional larval/adult association approaches, such as laboratory rearing and the metamorphotype approach of collecting mature pupae and associating larval sclerites and developed adult genitalia within [5]. As such, the larvae of the majority of North American Trichoptera species remain unknown.

There appears to be promise for Trichoptera larval identification through the use of DNA barcoding. In Trichoptera, the DNA barcode region – a mitochondrial cytochrome *c* oxidase subunit I (COI) fragment – is commonly employed as it has been found to have a low intraspecific variation and high interspecific divergence, or a barcoding gap, in most caddisfly species tested, which allows for clear species delineation based on genetic clustering [6-9]. This ability to correctly identify a specimen based on its DNA sequence is particularly useful for associating adult and immature stages of Trichoptera [5,10-14]. By matching an inquiry DNA sequence of an immature life stage to that of a morphologically identified adult, a species-level identification for the larva can be supplied [5]. In addition, the association of different life stages can help delimit morphologically vague species boundaries in adults, as was found in Chinese *Mexipsyche* (Hydropsychidae) species with distinct larval head markings but cryptic adult male genitalia [5], and in the *Diplectrona modesta* Banks, 1908 complex in North America [15].

However, the ability to reliably identify an immature life stage through a DNA sequence is dependent on a thorough reference library of DNA barcodes. A geographic area that has had substantial effort to compile a reference library is the subarctic region of Churchill, MB, Canada. Churchill is situated on the southern coast of the Hudson Bay, which marks the transition from boreal to tundra ecosystems, and has also been the focus of a collaborative effort to document the biodiversity of the region for a variety of taxonomic groups – the Polar Barcode of Life campaign (http://www.polarbarcoding.org). This multi-year effort reported 14 Trichoptera families, 32 genera, and 68 species in recent years [7,8], and built a DNA barcode reference library for the area, which can be accessed through the Barcode of Life Data Systems (BOLD, http://www.boldsystems.org) [16]. The linkage between a reference library of DNA barcodes and morphologically identified adult voucher specimens for Churchill has enabled rapid and reliable association of Trichoptera larvae with their adults for ecological applications.

The aim of this study was to supplement the current BOLD library of Trichoptera and to provide morphological diagnoses, photographs, and an appropriate taxonomic key for the Trichoptera larvae of the Churchill area. Additional collections and analyses since previous reports [7,8] has resulted in 23 additional taxa being collected from the Churchill area. DNA analysis for this material has resulted in morphological information to allow separation for nearly all of the collected Churchill caddisfly larvae. Development of the taxonomic characters was greatly expedited via use of known DNA associated material.

## Results

### An updated checklist for the caddisflies of Churchill

Since the first publications on the EPT faunas of Churchill in 2009 and 2010 [7,8], which included only adult sequences, an additional 1,810 caddisfly barcodes have been sequenced (a total of 3,310 COI barcodes being analyzed in the present study), including 148 adults and 1,662 larvae. These new sequences are deposited in BOLD projects: CUTRI, CUTLB, CUTLV, LBTLT, and EBTCH. GPS coordinates and habitat information are publically accessible on BOLD. All COI sequences are available on GenBank under accession numbers: GU680248-GU680333; GU680935-GU681016; GU681233-GU681319; GU711870-GU712502; HM398926-HM398969; HM421583; HM909539-HM909550; HQ944371; HQ962944; HQ986513-HQ986683; JF891300-JF891303; JX681817-JX682406; JX682408-JX682522. A total of 91 Trichoptera species (including provisional taxa) are reported in the present paper, including 23 new species records for the Churchill area.

### Larval-adult association

**Figure 1:**
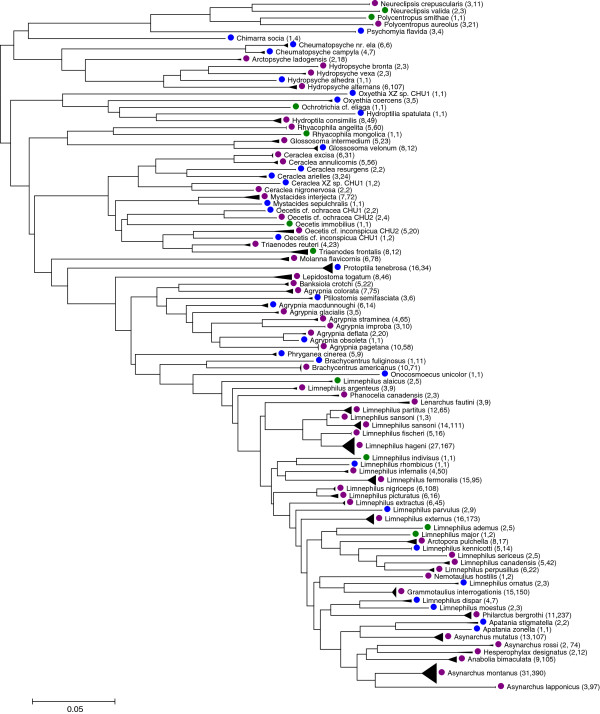
**Larval association of the Trichoptera species in the Churchill area.** Species represented by both adult and larval specimens were marked in purple color; those represented only by adults were marked in blue; and those by only larvae were marked by green.

Larvae of 62 species have been collected and associated in this work (Table 1, Figure 1), of which 11 taxa [*Ochrotrichia* cf. *eliaga*, *Oecetis immobilis* (Hagen, 1861), *Triaenodes frontalis* Banks, 1897, *Limnephilus ademus* Ross, 1941, *L*. *alaicus* (Martynov, 1915), *L*. *indivisus* Walker, 1851, *L*. *major* (Martynov, 1909), *Phanocelia canadensis* (Banks, 1924), *Neureclipsis valida* (Walker, 1852), *Polycentropus smithae* Denning, 1949, and *Rhyacophila mongolica* Levanidova, 1993] are represented by only larval specimens. These larvae were assigned to species based on additional barcode references available in BOLD through the Trichoptera Barcode of Life Campaign, using criteria described in the Methods.

**Table 1:** Adult and larval specimens collected from the Churchill area included in this study

**Family**		**Species**	**# Adults**	**# Larvae**
**Apataniidae**				
		*Apatania stigmatella* (Zetterstedt, 1840)	2	0
		*Apatania zonella* (Zetterstedt, 1840)	1	0
**Brachycentridae**				
		*Brachycentrus americanus* (Banks, 1899)	3	68
		*Brachycentrus fuliginosus* Walker, 1852	11	0
**Glossosomatidae**				
		*Glossosoma intermedium* (Klapálek, 1892)	15	8
		*Glossosoma velonum* Ross, 1938	12	0
		*Protoptila tenebrosa* (Walker, 1852)	34	0
**Hydropsychidae**				
		*Arctopsyche ladogensis* (Kolenati, 1859)	12	6
		*Cheumatopsyche campyla* Ross, 1938	7	0
		*Cheumatopsyche ela* Denning, 1942	2	0
		*Cheumatopsyche* nr. *ela*	4	0
		*Hydropsyche alhedra* Ross, 1939	1	0
		*Hydropsyche alternans* (Walker, 1852)	103	4
		*Hydropsyche bronta* Ross, 1938	2	1
		*Hydropsyche vexa* Ross, 1938	1	2
**Hydroptilidae**				
		*Hydroptila consimilis* Morton, 1905	39	10
		*Hydroptila spatulata* Morton, 1905	1	0
	*	*Ochrotrichia* cf. *eliaga*	0	1
	*	*Oxyethira* XZ sp. CHU1	1	0
		*Oxyethira coercens* Morton, 1905	5	0
**Lepidostomatidae**				
		*Lepidostoma togatum* (Hagen, 1861)	29	17
**Leptoceridae**				
		*Ceraclea annulicornis* (Stephens, 1836)	45	11
		*Ceraclea arielles* (Denning, 1942)	24	0
	*	*Ceraclea erratica* (Milne, 1936)	2	0
		*Ceraclea excisa* (Morton, 1904)	11	20
	*	*Ceraclea nigronervosa* (Retzius, 1783)	1	1
		*Ceraclea resurgens* (Walker, 1852)	2	0
		*Mystacides interjecta* (Banks, 1914)	11	62
		*Mystacides sepulchralis* (Walker, 1852)	1	0
		*Oecetis* cf. *inconspicua* CHU1	1	0
		*Oecetis* cf. *inconspicua* CHU2	20	1
		*Oecetis* cf. *ochracea* CHU1	2	0
		*Oecetis* cf. *ochracea* CHU2	3	4
	*	*Oecetis immobilis* (Hagen, 1861)	0	1
	*	*Triaenodes frontalis* Banks, 1907	0	12
		*Triaenodes reuteri* McLachlan, 1880	3	17
**Limnephilidae**				
		*Anabolia bimaculata* (Walker, 1852)	9	97
		*Arctopora pulchella* (Banks, 1908)	12	5
		*Asynarchus lapponicus* (Zetterstedt, 1840)	2	95
		*Asynarchus montanus* (Banks, 1907)	173	218
		*Asynarchus mutatus* (Hagen, 1861)	77	30
		*Asynarchus rossi* (Leonard & Leonard, 1949)	35	39
		*Grammotaulius interrogationis* (Zetterstedt, 1840)	119	32
	*	*Hesperophylax designatus* (Walker, 1852)	11	1
		*Lenarchus fautini* (Denning, 1949)	6	3
	*	*Limnephilus ademus* Ross, 1941	0	5
	*	*Limnephilus alaicus* (Martynov, 1915)	0	5
	*	*Limnephilus argenteus* Banks, 1914	3	6
		*Limnephilus canadensis* Banks, 1908	1	41
	*	*Limnephilus dispar* McLachlan, 1875	7	0
		*Limnephilus externus* Hagen, 1861	66	107
	*	*Limnephilus extractus* Walker, 1852	7	38
		*Limnephilus femoralis* Kirby, 1837	94	1
		*Limnephilus fischeri* Ruiter, 1995	13	3
		*Limnephilus hageni* Banks, 1930	122	45
	*	*Limnephilus indivisus* Walker, 1852	0	1
		*Limnephilus infernalis* (Banks, 1914)	17	34
		*Limnephilus kennicotti* Banks, 1920	14	0
	*	*Limnephilus major* (Martynov, 1909)	0	2
		*Limnephilus moestus* Banks, 1908	3	0
		*Limnephilus nigriceps* (Zetterstedt, 1840)	5	102
	*	*Limnephilus ornatus* Banks, 1897	3	0
		*Limnephilus partitus* Walker, 1852	12	53
	*	*Limnephilus parvulus* (Banks, 1905)	9	0
		*Limnephilus perpusillus* Walker, 1852	8	14
		*Limnephilus picturatus* McLachlan, 1875	6	10
		*Limnephilus rhombicus* Linnaeus, 1758	1	0
		*Limnephilus sansoni* Banks, 1918	87	28
		*Limnephilus sericeus* (Say, 1824)	4	1
	*	*Nemotaulius hostilis* (Hagen, 1873)	1	1
		*Onocosmoecus unicolor* (Banks, 1897)	1	0
	*	*Phanocelia canadensis* (Banks, 1924)	0	3
		*Philarctus bergrothi* McLachlan, 1880	6	230
**Molannidae**				
		*Molanna flavicornis* Banks, 1914	45	33
**Philopotamidae**				
		*Chimarra socia* Hagen, 1861	4	0
**Phryganeidae**				
		*Agrypnia colorata* Hagen, 1873	72	3
		*Agrypnia deflata* (Milne, 1931)	15	5
	*	*Agrypnia glacialis* Hagen, 1873	4	1
		*Agrypnia improba* (Hagen, 1873)	7	3
		*Agrypnia macdunnoughi* (Milne, 1931)	14	0
		*Agrypnia obsoleta* (Hagen, 1864)	1	0
	*	*Agrypnia pagetana* Curtis, 1835	13	45
		*Agrypnia straminea* Hagen, 1873	35	30
		*Banksiola crotchi* Banks, 1943	21	1
		*Phryganea cinerea* Walker, 1852	9	0
		*Ptilostomis semifasciata* (Say, 1828)	6	0
**Polycentropodidae**				
		*Neureclipsis crepuscularis* (Walker, 1852)	4	7
	*	*Neureclipsis valida* (Walker, 1852)	0	3
		*Polycentropus aureolus* (Banks, 1930)	4	17
	*	*Polycentropus smithae* Denning, 1949	0	1
**Psychomyiidae**				
		*Psychomyia flavida* Hagen, 1861	4	0
**Rhyacophilidae**				
		*Rhyacophila angelita* Banks, 1911	41	20
	*	*Rhyacophila mongolica* Schmid, Arefina & Levanidova, 1993	0	1
				
**Trichoptera Total**			**1644**	**1666**

New records indicated with an asterisk (*).

### Synoptic discussion of Churchill Trichoptera larvae

#### Apataniidae

Adults of two *Apatania* species [*A. stigmatella* (Zetterstedt, 1840) and *A*. *zonella* (Zetterstedt, 1840)] have been collected although no larvae were found. Larval descriptions for both taxa are available in Lepneva [17] and Solem [18], although Solem indicated his specimens varied from those of Lepneva. Based on Solem [18], it is possible the Churchill taxa are separable based on the shape of the metanotal sa1 area: *A*. *stigmatella* with two separate setal areas; *A*. *zonella* with a single, contiguous setal area.

#### Brachycentridae

**Figure 2:**
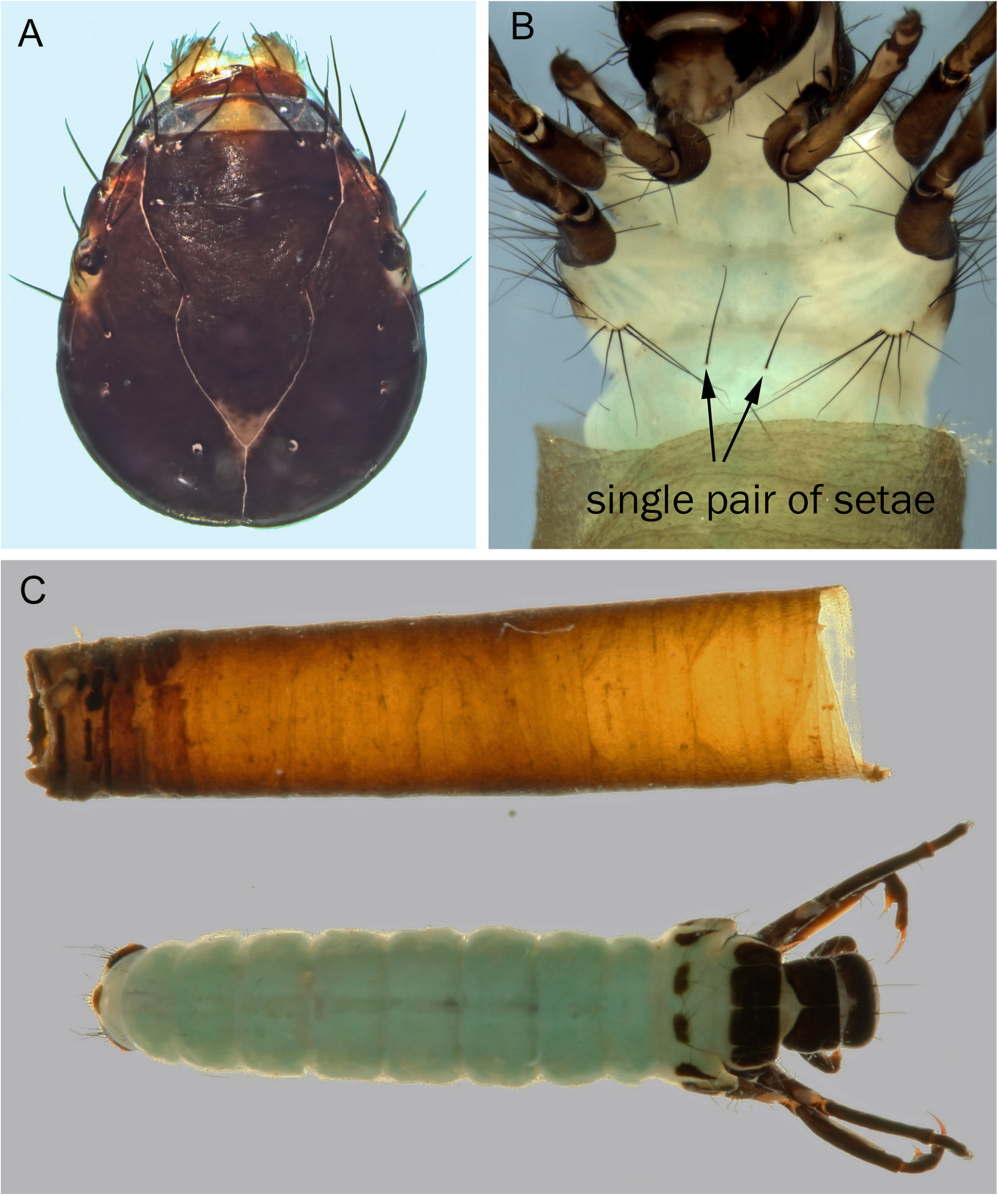
***Brachycentrus americanus *A: head, dorsal; B: abdomen, ventral; C: habitus and case, dorsal.**

Two brachycentrid species, *Brachycentrus americanus* (Banks, 1899) (Figure 2) and *B*. *fuliginosus* Walker, 1852, have been collected in the Churchill area. To date, only larvae of the former species have been collected in the Churchill area. The combination of Flint [19] and Harrington and Morse [20] provide excellent descriptions for all the North American *Brachycentrus* larvae except *B*. *fuliginosus*, which remains unknown.

In the Churchill area, *Brachycentrus* larvae are readily identified based on the case, which is usually square, although several Churchill area collections have cases that are partially round and composed of silk (Figure 2C). While the larva of *B*. *fuliginosus* is currently unknown, Flint [19] placed *B. fuliginosus* in the subgenus *Sphinctogaster* and *B*. *americanus* in the subgenus *Brachycentrus* based on adult characters. Flint also provided a hindfemur setal character to separate the two subgenera: the dorsal hindfemoral setae of *Brachycentrus* are limited to two strong setae while those of *Sphinctogaster* are more abundant and weaker. *Sphinctogaster* should also have two pairs of setae located on the venter of the first abdominal segment. A single pair occurs in *B*. *americanus.*

#### Glossosomatidae

While three glossosomatid taxa have been collected as adults (Table 1) in the Churchill area, only larvae of *Glossosoma intermedium* (Kapalek, 1892) were associated via their DNA sequences (see figures below for *Glossosoma intermedium*, lateral). Wiggins [21] provides characters to separate the *Glossosoma* from the *Protoptila*, however we are unaware of larval morphological characters to separate the North American species of *Glossosoma* or *Protoptila*.

#### Hydropsychidae

**Figure 3:**
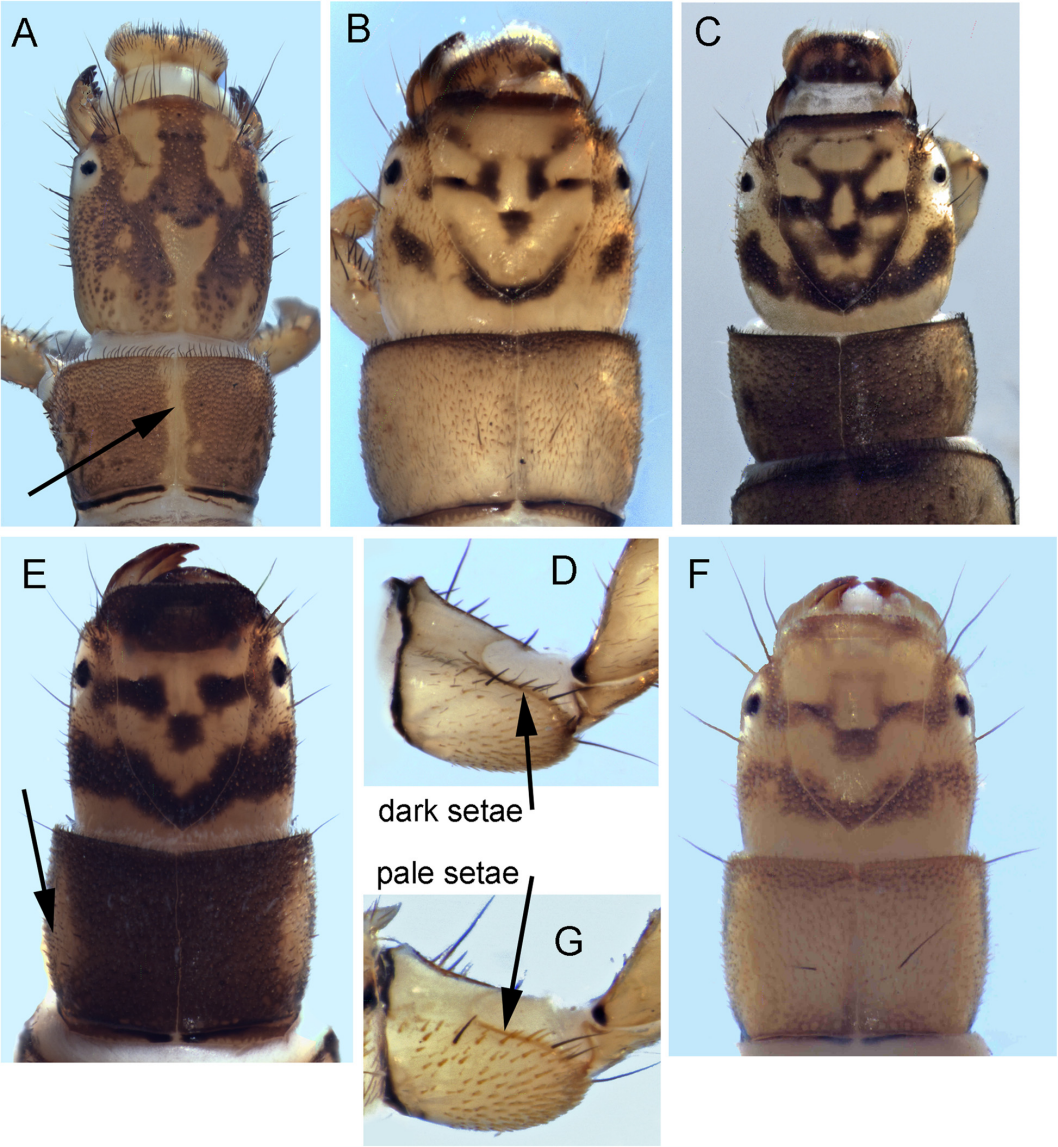
***Arctopsyche ladogensis *A: head, dorsal; *Hydropsyche alternans *B & C: color variation, dorsal, D: procoxa; *Hydropsyche bronta *E: head, dorsal; *Hydropsyche vexa *F: head dorsal, G: procoxa.**

Eight hydropsychids have been found in the Churchill area, including the *Cheumatopsyche campyla* Ross, 1938 complex, which may be represented by 3 cryptic lineages (*C*. *campyla*, *C*. *ela* Denning, 1942, and *C*. nr. *ela*), for which larvae are unavailable. Larvae of 4 hydropsychid species have been associated (Table 1, Figure 3).

*Arctopsyche ladogensis* (Kolenati, 1859) larvae have a pale midline stripe continuing from the head to the thorax (Figure 3A). The head is long relative to *Hydropsyche* and the anterior margin of the frontoclypeus is convex rather than squared off as in *Hydropsyche*.

The head patterns of the Churchill area *Hydropsyche* are fairly distinctive. However, these larval characters have been shown to be highly variable within *Hydropsyche* species [22,23]. This is also evident in Churchill specimens (Figure 3B-C). Schefter and Wiggins [23] provided morphological characters for separation of the three known Churchill *Hydropsyche* species, which should be examined if additional color variations are found.

*Hydropsyche alternans* (Walker, 1852) has the checkerboard head pattern, although based on the two Churchill specimens it can appear quite different between light and dark specimens. *H. alternans* can be separated from *H. vexa* Ross, 1938 by the presence of dark setae along the dorsal margin of the procoxal outer surface (Figure 3D).

*H. bronta* Ross, 1938 larvae (based on a single Churchill specimen) have a two-toned pronotum (Figure 3E), which is pale laterally and darkened dorsally. This character is distinct from the other two known Churchill area *Hydropsyche*.

The pronotum of *H*. *vexa* is pale yellow without obvious markings other than the anterior and posterior darkening evident on some specimens (Figure 3F). The head has a checkerboard pattern. It can be separated from *H*. *alternans* by the presence of pale setae along the dorsal margin of the procoxal outer surface (Figure 3G).

Lepneva [24] provided a detailed description of the *Arctopsyche ladogensis* larvae. Schuster and Etnier [22] provided a description for *H*. *bronta* and Schefter and Wiggins [23] described all four Churchill area *Hydropsyche* larvae, although we did not collect larval *H*. *alhedra*.

#### Hydroptilidae

**Figure 4:**
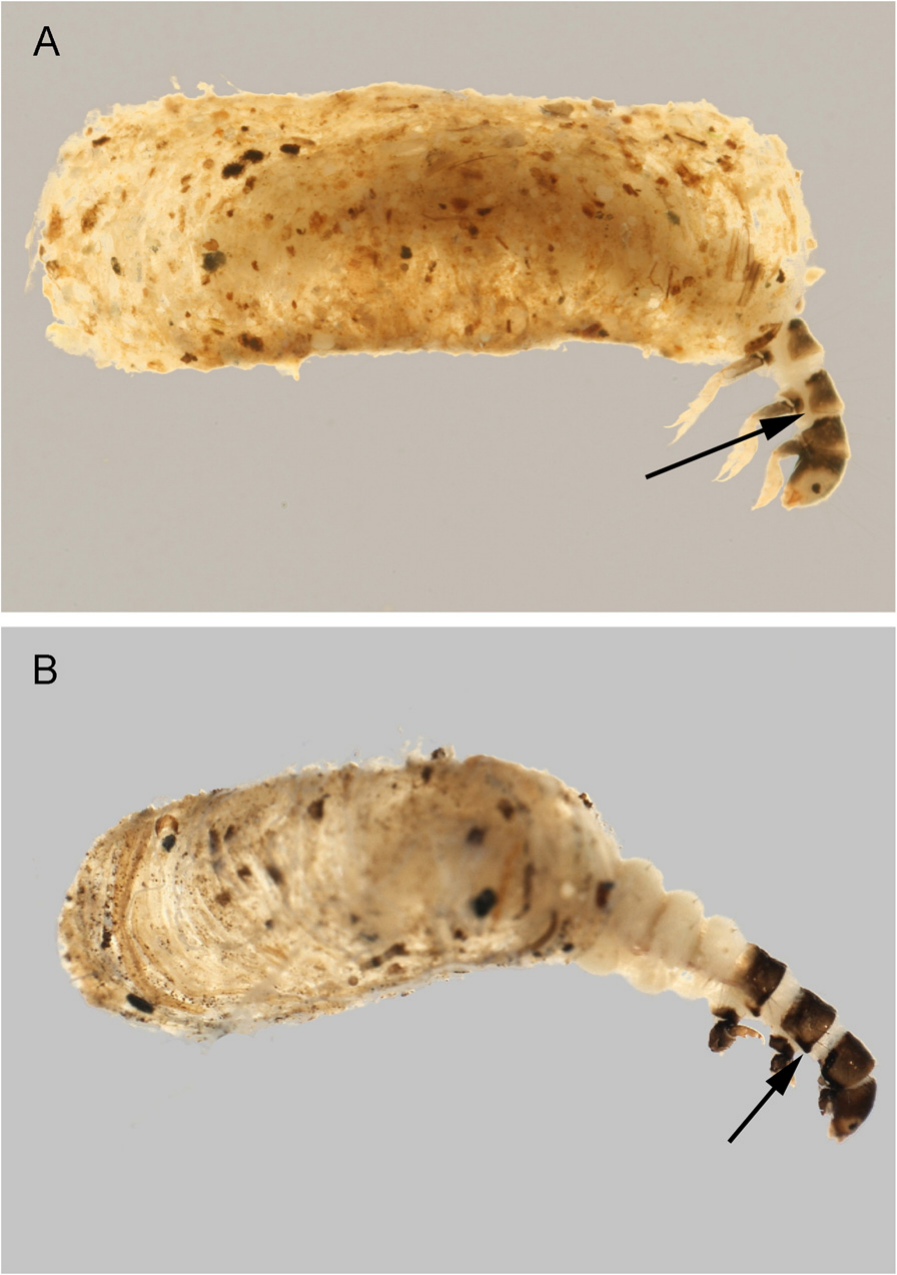
***Hydroptila consimilis *A: lateral; *Ochrotrichia *cf. *eliaga *B: lateral.**

Of the five hydroptilid taxa collected in the Churchill area (Table 1), only 2 larvae [*Hydroptila consimilis* Morton, 1905 and *Ochrotricha* cf. *eliaga* (a single specimen)] have been collected. These two genera have very similar larvae [21] and, in the Churchill specimens, can be most readily separated by the presence of a dark, elongated, anteroventral lobe on the mesonotum in *Ochrotrichia*, which is lacking in *Hydroptila* (Figure 4). Other characters to separate larvae of these two genera are also available [21]. We are unaware of larval descriptions for these two species.

#### Lepidostomatidae

**Figure 5:**
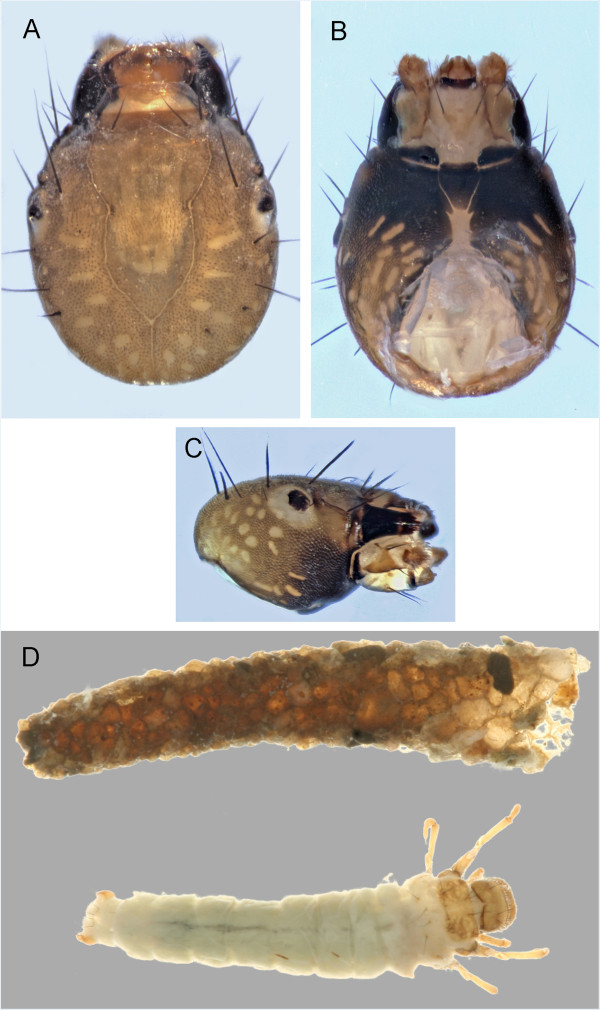
***Lepidostoma togatum *A: head, dorsal; B: head, ventral; C: head, lateral; D: habitus, dorsal.**

A single species of *Lepidostoma* [*L*. *togatum* (Hagen, 1861)] has been collected in the Churchill area. Weaver [25] pointed out that *L*. *togatum* is one of the most wide ranging *Lepidostoma* in North America and the panel-cased larvae inhabit both lotic and lentic habitats. In the Churchill area, this species is primarily lotic and its case is composed of fine mineral grains arranged in a smooth, tapered, slightly curved manner (Figure 5). The dorsum of the head has pale muscle scars on a darker background, typical of many *Lepidostoma*. The venter of the head is much darker anteriorly than the dorsum, with a few pale, linear muscle scars posterolaterally.

#### Leptoceridae

Based on morphological examination and barcode analysis, 15 leptocerid taxa have been collected in the Churchill area (Table 1, Figures 1). *Oecetis inconspicua* (Walker, 1852) and *O*. *ochracea* Curtis, 1825 each forms two distinct COI clusters, which are supported by genitalic morphological features [8]. Larvae of only 1 of each of these species pairs were collected and examined in this study. Provisional taxon codes from Zhou et al. [8] are followed here.

The 9 larval leptocerid taxa associated via DNA from the Churchill area include: *Ceraclea annulicornis* (Stephens, 1836), *C. excisa* (Morton, 1904), *C. nigronervosa* (Retzius, 1783) (1 specimen), *Mystacides interjecta* (Banks, 1914), *Oecetis immobilis* (1 specimen), *O*. cf. *inconspicua* CHU1 (1 specimen), *O*. cf. *ochracea* CHU2 (1 specimen), *Triaenodes frontalis*, and *T*. *reuteri* McLachlan, 1880. A combination of larval head coloration and setation can be used to separate all 9 taxa.

**Figure 6:**
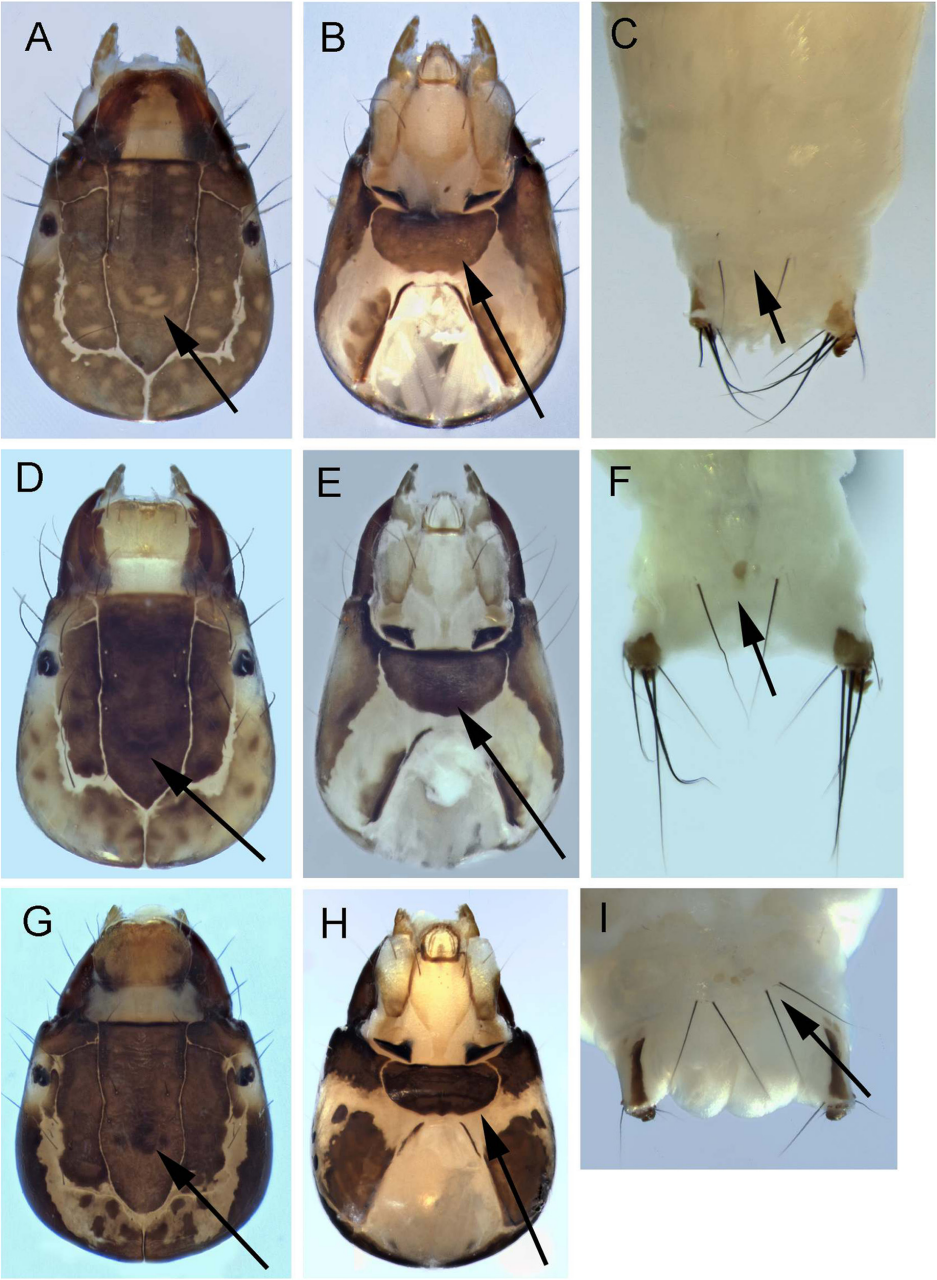
***Ceraclea annulicornis *A: head dorsal; B: head ventral; C: 9**^**th**^** tergite dorsal; *Ceraclea excisa *D: head dorsal; E: head ventral; F: 9**^**th**^** tergite dorsal; *Ceraclea nigronervosa *G: head dorsal; H: head ventral; I: 9**^**th**^** tergite dorsal.**

The three Churchill *Ceraclea* larvae that were associated by COI (*C. annulicornis, C. excisa, C. nigronervosa*) can be separated by a combination of head coloration and 9^th^ tergite setation (Figure 6). Resh [26] provided life histories and descriptions for 23 North American species and included all but *C*. *erratica* from the Churchill *Ceraclea* taxa.

*Ceraclea annulicornis* can be distinguished from the other known Churchill *Ceraclea* by the combination of the dark frontoclypeal area with pale muscle scars and a single pair of long setae on the 9^th^ tergite (Figures 6A&C). The case is made of small mineral grains with the dorsal anterior margin extended and curved downward over the anterior entrance.

*Ceraclea excisa* has the single pair of long setae on the 9^th^ tergite like *C*. *annulicornis*. It can be distinguished from the latter by the dark muscle scars on the dark frontoclypeus (Figures 6D&F). The mineral case is more narrowly tapered than that of *C*. *annulicornis* and the dorsal anterior area only slightly overhangs the case opening.

*Ceraclea nigronervosa* can be separated from the other Churchill *Ceraclea* by the presence of 2 pairs of setae on the 9^th^ tergite (Figure 6I). The frontoclypeus has dark muscle scars on a dark background and the case is comprised primarily of silk with attached detritus.

**Figure 7:**
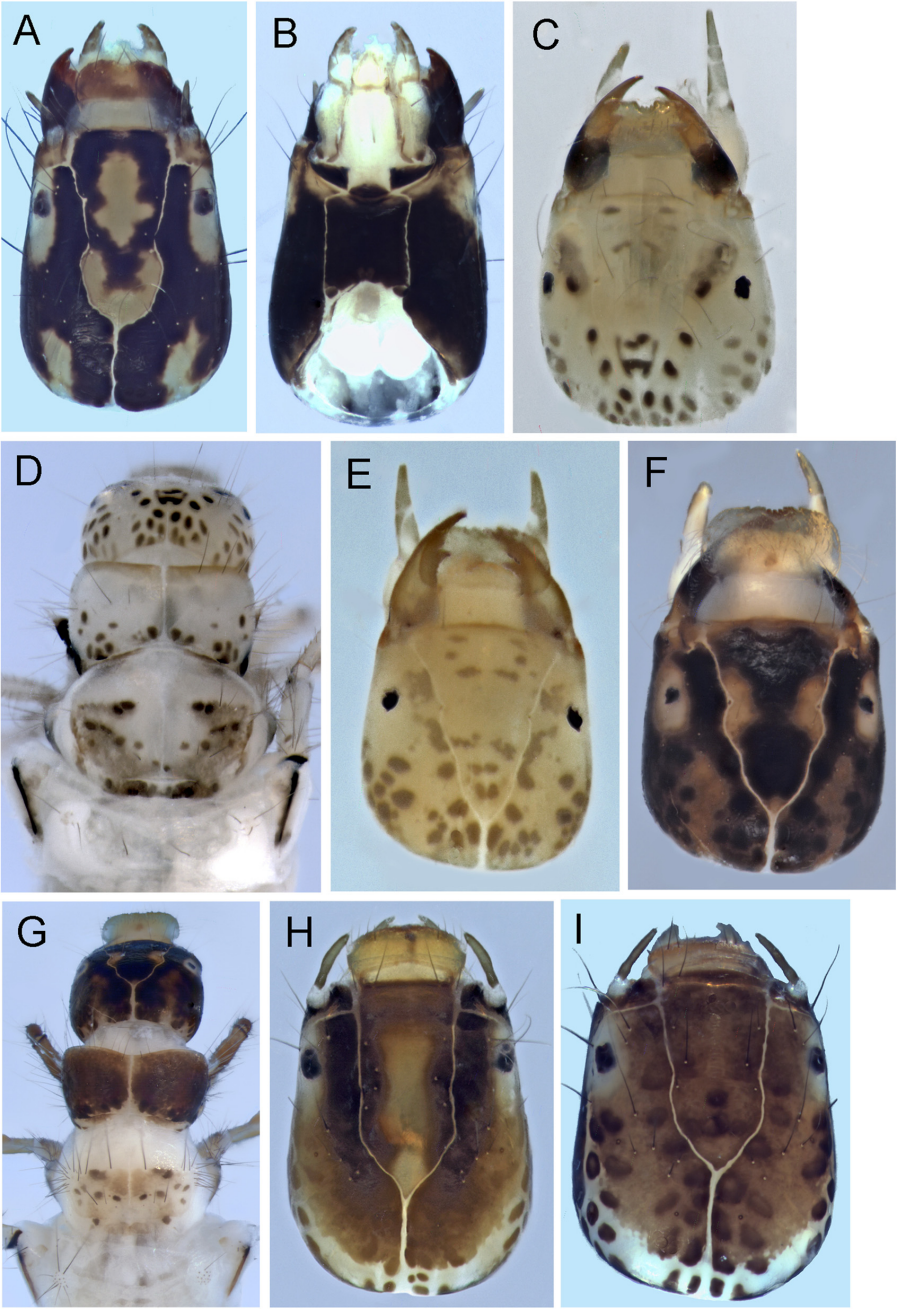
***Mystacides interjecta *A: head, dorsal; B: head, ventral; *Oecetis immobilis *C: head, dorsal, D: thorax, dorsal; *Oecetis *cf. *inconspicua *CHU1 E: head, dorsal; *Oecetis *cf. *ochracea *CHU2 F: head, dorsal; G: thorax, dorsal; *Triaenodes frontalis *H: head, dorsal; *Triaenodes reuteri*: I: head, dorsal.**

Larvae of both Churchill *Mystacides* have been described [27]. *M. interjecta* has a straight case made of sand grains and small bark particles (see figures below for *M. interjecta*). They lack the large, long, ballast sticks typical of *Mystacides*, although the head color pattern is the same as portrayed by Yamamoto and Wiggins [27] (Figure 7A). *M. sepulchralis* (Walker, 1852) larvae were not collected from the Churchill area during this work.

Based on DNA and morphology, possibly five distinct *Oecetis* species have been collected in the Churchill area [8]. Two distinct genetic lineages of *Oecetis* cf. *inconspicua*, two of *O*. cf. *ochracea* and *O*. *immobilis* were collected. Floyd [28] pointed out the difficulty with the morphological separation of *Oecetis* larvae and the Churchill DNA results support that conclusion. Very few of the Churchill *Oecetis* larvae were collected: one each for *O*. *immobilis* and *O*. cf. *inconspicua* CHU1, and only three for *O.* cf. *ochracea* CHU2.

*Oecetis immobilis* can be separated from the other known Churchill *Oecetis* larvae by the combination of pale head background color and a large, dark blotch between the eye and frontoclypeus (Figure 7C). The single Churchill specimen is immature and the colors are not fully developed although it is similar to the figures of Floyd [28].

*Oecetis* cf. *inconspicua* CHU1 is very similar to *O. immobilis* but differs in the scattered dark muscle scars arranged along the frontoclypeal suture behind the eye, which are absent in *O. immobilis* (Figure 7E). These characters are also based on a single immature specimen and do not match any of the suspected *O. inconspicua* included in Floyd [28].

*Oecetis* cf. *ochracea* CHU2 is readily separated from the other known Churchill *Oecetis* by the dark base color (Figure 7F). It also does not match the description by Floyd [28].

There are two *Triaenodes* in the Churchill area: *T*. *frontalis* and *T*. *reuteri*. They both construct spiral plant cases (see figures for *T. frontalis* and *T.reuteri* below). The larvae can be separated by the head patterns (Figure 7H&I). Glover [29] discussed (as *Ylodes*) several additional unknown larvae related to *T. frontalis* and *T. reuteri*. *T*. *frontalis* have a pale, medial stripe and no muscle scars on the frontoclypeus. It is similar to the *T*. *frontalis* description of Glover [29]. *T*. *reuteri* has a dark background on the frontoclypeus and numerous, darker muscle scars, which is also similar to the description by Glover [29].

#### Limnephilidae

**Figure 8:**
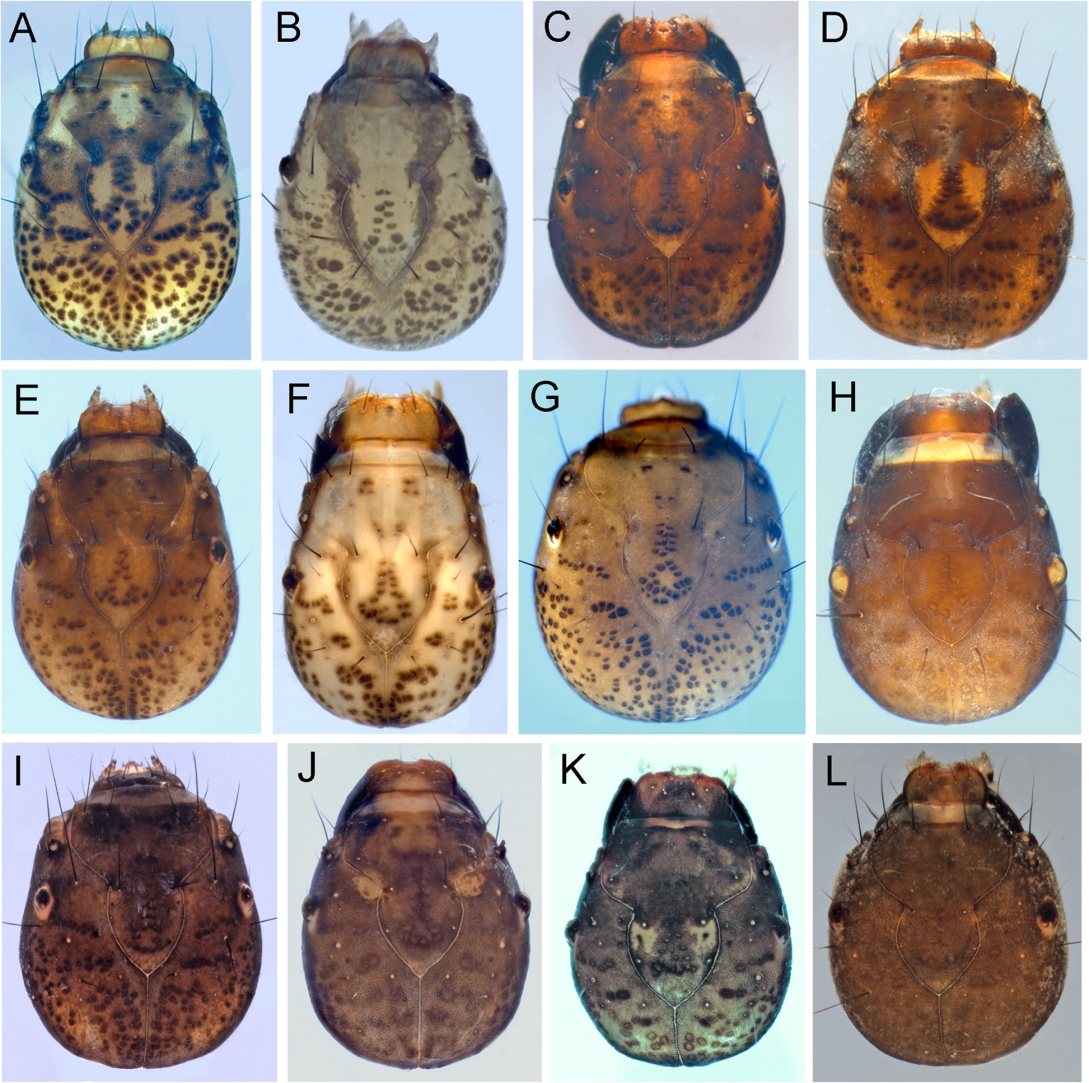
**Limnephilidae heads, dorsal view A: *Anabolia bimaculata; *B: *Arctopora pulchella; *C: *Asynarchus lapponicus; *D: *Asynarchus montanus; *E: *Asynarchus mutatus; *F: *Asynarchus rossi; *G: *Grammotaulius interrogationis; *H: *Hesperophylax designatus; *I: *Lenarchus fautini; *J: *Limnephilus ademus; *K: *Limnephilus alaicus; *L: *Limnephilus argenteus.***

**Figure 9:**
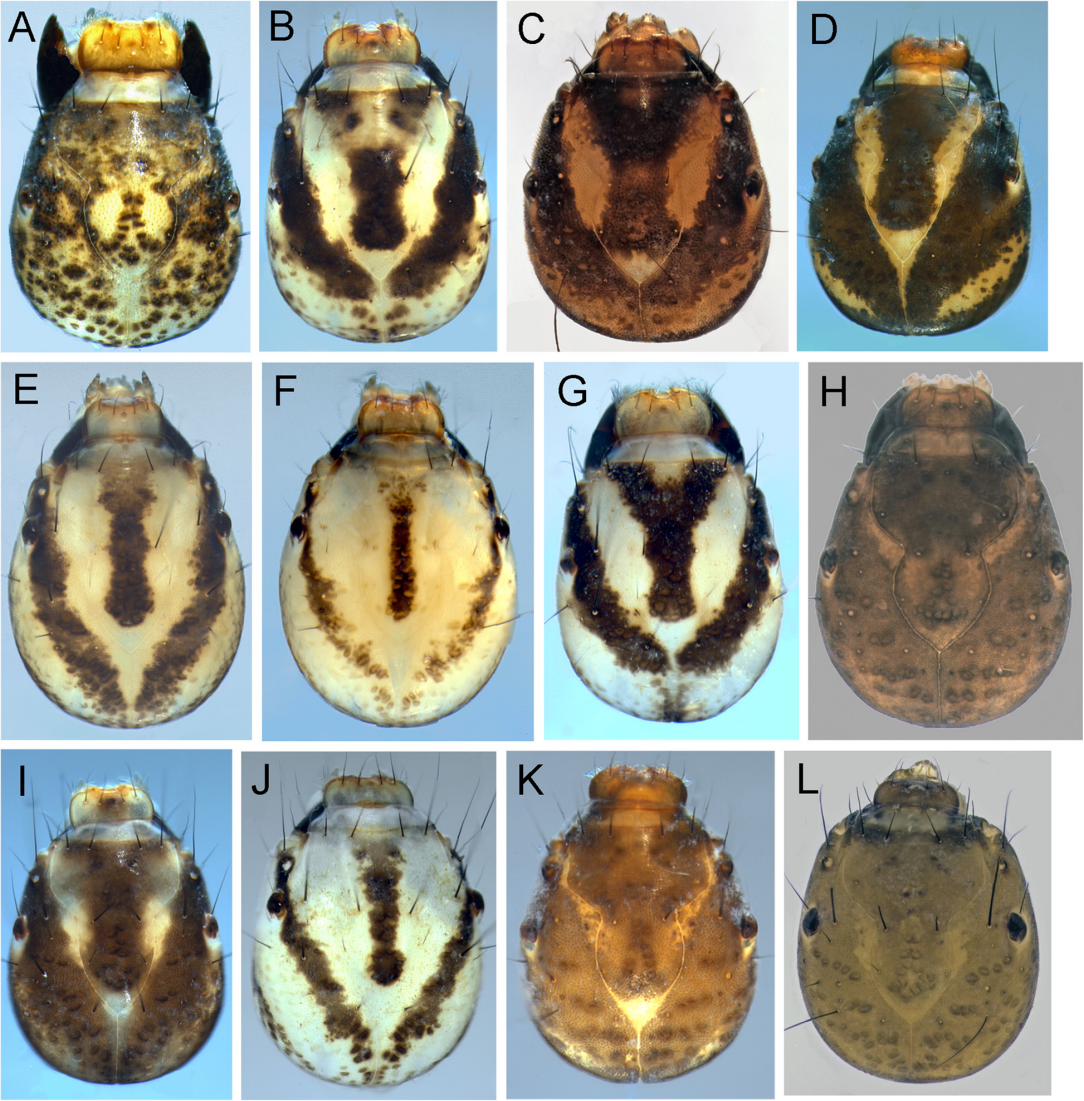
**Limnephilidae heads, dorsal view A: *Limnephilus canadensis; *B: *Limnephilus externus; *C: *Limnephilus extractus; *D: *Limnephilus femoralis; *E: *Limnephilus fischeri; *F: *Limnephilus hageni; *G: *Limnephilus infernalis; *H: *Limnephilus major; *I: *Limnephilus nigriceps; *J: *Limnephilus partitus; *K: *Limnephilus perpusillus; *L: *Limnephilus picturatus.***

**Figure 10:**
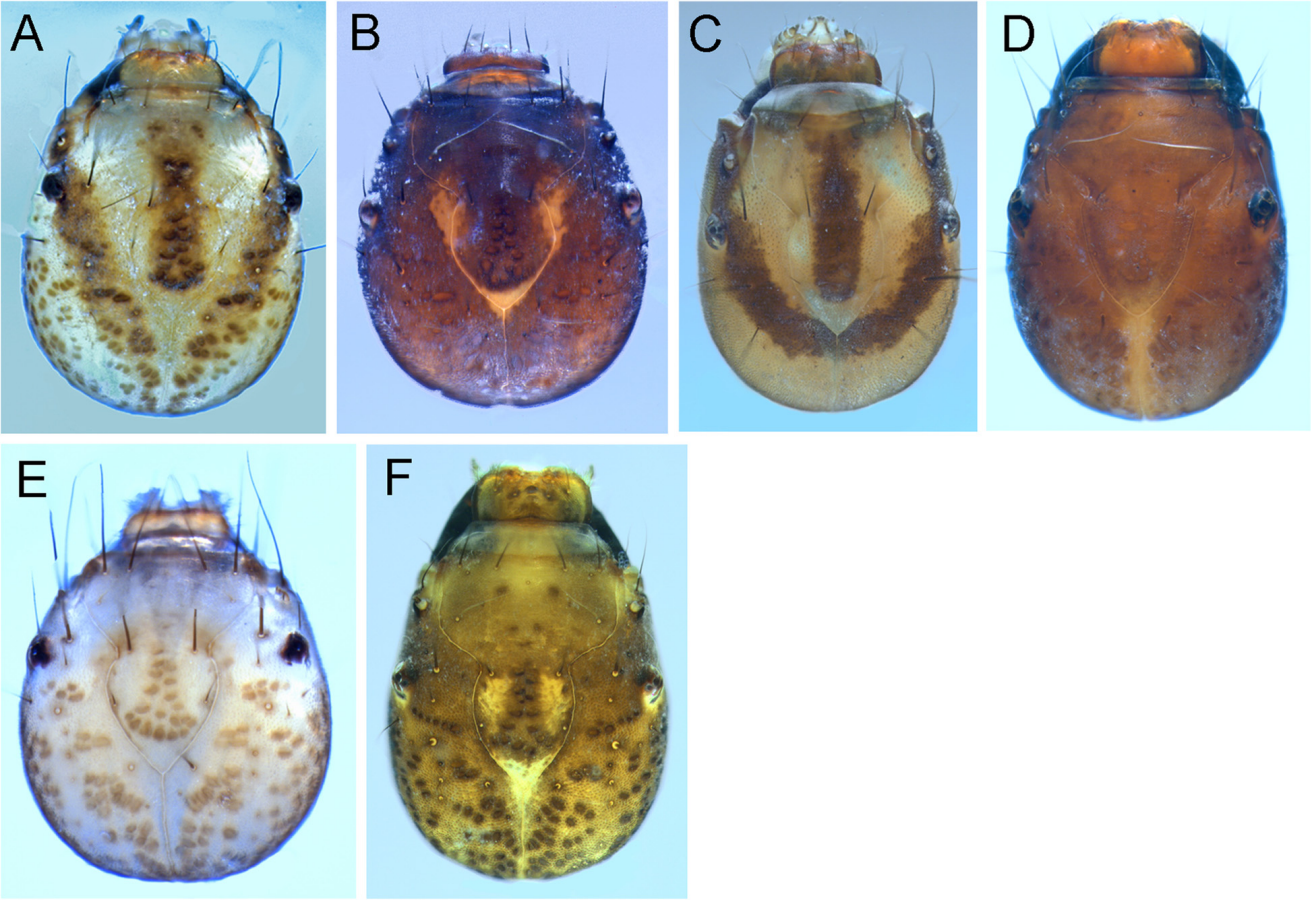
**Limnephilidae heads, dorsal view A: *Limnephilus sansoni; *B: *Limnephilus sericeus; *C: *Nemotaulius hostilus; *D: *Onocosmoecus unicolor; *E: *Phanocelia canadensis; *F: *Philarctus bergrothi.***

The limnephilids are the most diverse family found in the Churchill area. We have made larval associations for 23 of the 32 taxa. While highly diverse, the larvae of this small collection seem to be distinguishable based on a combination of head color patterns and various setal characters (Figures 8, 9, 10, 15, 16 and 17). Since there are a large number of limnephilid species in the Churchill area, it must be remembered that the discussion below, and the key at the end of this document, is primarily based on only one or two specimens of each species. Usually those specimens were missing at least one leg, and often more from routine barcoding analysis. Not all specimens were in good condition or of the last instar. So the characters presented here should be re-examined in detail when additional material becomes available. We have included observations of case characteristics for several of the species but it should be recognized that Limnephilidae cases, even within a single population, can be very different, and some species rebuild a completely different pupal case in the 5^th^ instar from that of the earlier instars.

##### Anabolia

A single species of this genus, *Anabolia bimaculata* (Walker, 1852), has been collected. The larval head and thorax have a pale yellow background color with numerous, distinct, dark muscle scars on both head and thoracic segments (Figure 8A); a single mesonotal sa1 seta, and dorsal abdominal chloride epithelia [21]. *Anabolia* also have a scurf of small, stout spicules on the anterolateral surface of the pronotum which is readily visible at 40X; and, accessory setae on the lateral surfaces of the meso- and metatrochanters and femur. The case, although variable, is always of vegetation. The larva of *A. bimaculata* has been described by Betten [30], Denning [31], and Flint [32].

##### Arctopora

A couple *Arctopora pulchella* (Banks, 1908) larval specimens were collected in the Churchill area (Figure 8B). The genus could be readily confused with *Anabolia* and *Grammotaulius* based on the case. The colors and muscle scars of *Arctopora* larvae tend to be paler and less distinct than *Anabolia* (Figure 8A). *Arctopora* lacks both the scurf of small, stout spicules on the anterolateral surface of the pronotum, and the accessory setae on the mesofemur lateral surfaces typical of *Anabolia*. *Arctopora* can be readily separated from *Grammotaulius* by the short ventral apotome, which does not extend to the posterior margin of the head. The two major ventral femoral spines of all three *Arctopora* legs are pale. The case is of vegetation and the larva was described (as *Lenarchulus*) by Flint [32].

##### Asynarchus

Larvae for all 4 species of this genus in the Churchill area have been associated. Their larvae can be separated from other limnephilid genera by a combination of: presence of accessory setae on the mesofemur lateral surface; scurf of small, stout spicules on the anterolateral surface of the pronotum absent; and [except for *A*. *rossi* (Leonard and Leonard, 1949)] presence of dorsal chloride epithelia. The cases tend to be fairly straight and composed of small mineral particles with occasional small vegetal particles. It appears larvae of the Churchill *Asynarchus* [unlike *Asynarchus contumax* (McLachlan, 1880) – see Solem [18]] convert their case to at least partial mineral material just prior to pupation (see Wiggins [21]). Flint [32] also reported vegetal pupal cases for species in this genus.

*Asynarchus lapponicus* Zetterstedt, 1840 (Figure 8C) is very similar to *A. montanus* (Banks, 1907)*.* Both have accessory setae on the basal trochantal segment. The gill character is based on examination of two 5^th^ instar larvae of both species. The abdominal ventrolateral gill row of *A. lapponicus* ends on the 4^th^ segment.

*Asynarchus montanus* larvae (Figure 8D) are distinguished from *A. lapponicus* (see discussion above) by the ventrolateral gill row ending on the 6^th^ or 7^th^ abdominal segment.

*Asynarchus mutatus* (Hagen, 1861) (Figure 8E) can be separated from *A*. *lapponicus* and *A*. *montanus* by the lack of accessory setae on the basal trochantal segment. Its ventrolateral abdominal gill series also extends to the 6^th^ or 7^th^ segment (n = 2).

*Asynarchus rossi* has been variously placed in *Asynarchus*[33,34] and *Limnephilus* (Leonard and Leonard 1949, Wiggins [21] - with Schmid’s concurrence) and presents interesting larval and adult characters. The placement of *A*. *rossi* on the Neighbor Joining tree was very far from the rest of the *Asynarchus* species as well as *Limnephilus* spp., suggesting a generic revision of the species is needed, although the NJ tree should not be inferred as a phylogenetic relationship for the relevant species. The distinctiveness of *A*. *rossi* is also supported by a phylogenetic tree built based on COI and 3 nuclear genes using a Bayesian approach (Boyle, unpublished data). The larvae have several setae on the metanotal membrane between the sa2 sclerites, which are absent in the other *Asynarchus*, and *Limnephilus*. Like *Limnephilus*, *A*. *rossi* lacks dorsal chloride epithelia. *A. rossi* larvae have a very pale yellow head and thorax background color, and, as a result lack the obvious 3-spot frontoclypeus (Figure 8F). In hand, the larvae appear to have a distinct dark, medial thoracic/head stripe. The case is normally comprised of mineral particles and is more curved than that of *Asynarchus* and *Limnephilus*.

##### Grammotaulius

As Wiggins [21] pointed out, the larvae of this genus seem to be highly variable. The larvae of the single Churchill species, *Grammotaulius interrogationis* (Zetterstedt, 1840), are most similar to *Arctopora pulchella* with base color and muscle scars of *Grammotaulius* larvae being very pale yellow with numerous dark muscle scars (Figure 8G). *G*. *interrogationis* also lacks the scurf of small, stout spicules on the anterolateral surface of the pronotum; lacks the accessory setae on the mesofemur lateral surfaces and have dorsal chloride epithelia. The case is of vegetation (see Wiggins [21]). *Grammotaulius* can be distinguished from *Arctopora* by the long ventral apotome, which reaches the posterior margin of the head. *Arctopora* is also smaller. When mature, *G*. *interrogationis* larvae exceed 25 mm in length while those of *A*. *pulchella* do not exceed 20 mm.

##### Hesperophylax

The only *Hesperophylax* species collected from the Churchill area is *H. designatus* (Walker, 1852) (Figure 8H), and is one of the three Churchill genera (*Lenarchus*, *Onocosmoecus*) with more than 3 gill filaments per cluster on some segments. It can be readily separated from *Lenarchus* and *Onocosmoecus* by the presence of accessory setae on the mesofemur lateral surfaces and on the metanotal surface between the sa2 sclerites. Flint [32] pointed out that the larvae of this species have probably been described more times than any other North American caddisfly species.

##### Lenarchus

We collected one *Lenarchus* species, *L. fautini* (Denning, 1949) (Figure 8I)*,* in the Churchill area. The larva has at least some gill clusters with more than six filaments (likely some clusters will have many more than 6 filaments) and usually a vegetation case made of large pieces like *Arctopora*, *Grammotaulius* and some *Anabolia*. *Lenarchus fautini* lack the setae located on the membrane between the metanotal sa2 sclerites, which are present in *Hesperophylax*. And at least the anterior gill clusters have many more gill filaments than the maximum of four or so in *Onocosmoecus unicolor* (Banks, 1897).

##### Limnephilus

*Limnephilus* larvae currently comprise a large, poorly defined genus. This uncertainty in phylogeny is very apparent in the Churchill *Limnephilus* larvae. Of the 24 *Limnephilus* species collected at Churchill, larvae of 18 have been associated via DNA. Over the years, major advancements in our ability to separate the North American *Limnephilus sensu lato* larvae have been largely limited to those of Lloyd [35], Flint [32] and Hoopes [36]. The most recent work of Wiggins [21,37] has greatly improved our ability to separate the *Limnephilus sensu lato* larvae from the other limnephilid genera although he pointed out that larvae of only 5 North American *Limnephilus* species had been described at the time. More frequent advances in Holarctic larval *Limnephilus* taxonomy have occurred, particularly with the work of Lepneva [17], Hiley [38], Wallace et al. [39] and Waringer & Graf [40]. The recent Wallace et al. [39] publication provides the best summary available today of characters useful for species determinations.

*Limnephilus ademus* - The larvae of *L. ademus* belong to the group with both: a dark base colored head with darker muscle scars (Figure 8J); and, accessory setae present on the lateral margins of the meso- and metafemur. The head has two pale blotches primarily outside the anterior constriction of the frontoclypeal suture. A slight pale area is also present in the posterior apex of the frontoclypeal suture. It is most similar to *L. major*, another member of the *L. incisus* group. The larva was described by Flint and Giberson [41] although the figure of the head presented there does not show the pale areas anterior of the eyes shown here, which occurs in the Churchill larvae.

*Limnephilus alaicus* - The larvae of *L. alaicus* is another of the group with accessory setae on the lateral margins of the meso- and metafemur. It can be separated from the closely related *L. incisus* group larva by the anterior pale areas of the head, which are primarily located within the frontoclypeal sutures (Figure 8K). Grigorenko [42] synonymized *L. alaicus, L. pallens* (Banks, 1920) (a North American species), and *L. tricalcaratus* (Mosely, 1936) under *L. samoedus* (McLachlan, 1880). Malicky [43] resurrected *L. alaicus*. It will take further DNA and morphological studies of all taxa within the group to determine which species are valid.

*Limnephilus argenteus* Banks, 1914 larvae belong to the group of Churchill *Limnephilus* with the character combination of: head with a dark base color and darker muscle scars (Figure 8L); and, lateral margins of the meso- and metafemur lacking accessory setae. Based on the examined specimens, *L. argenteus* can be separated from the other Churchill larvae in this group [*L. perpusillus* Walker, 1852*, L. picturatus* McLachlan, 1875*, L. sericeus* (Say, 1824)] by the very monochromatic brown head coloration with little evidence of pale blotches in the frontoclypeal area.

*Limnephilus canadensis* Banks, 1908 larvae are relatively small, less than 15 mm in length. And the cases are made of medium size mineral particles. There are both dark and light large, primary meso- and metafemur ventral setae with the distal one of the pair pale on the mesofemur and the proximal one pale on the metafemur. The head and thorax have a pale yellow background color with many dark muscle scars (Figure 9A).

*Limnephilus externus* Walker, 1852 has a distinctive prothoracic color pattern with a large dark band along the anterior margin. The head pattern (Figure 9B) is essentially the same as *Nemotaulius hostilus* Hagen, 1861. The two primary setae along the ventral meso- and metafemur margins are dark. It is very similar to *L. extractus* Walker, 1852 but can be separated by the presence of numerous setae at the mesonotal sa1 location.

*Limnephilus extractus* is another taxa with the characteristic three-band head pattern (Figure 9C) and wide dark band along the anterior margin of the pronotum. It has a single seta at the mesonotal sa1 position (see discussion at *L. externus*)*.*

*Limnephilus femoralis* (Zetterstedt, 1840) is one of the four Churchill area taxa with the characteristic three-band head pattern (Figure 9D) and wide dark band along the anterior margin of the pronotum. We had only a single larva for examination. The lateral dark head bands extend to the hind margin of the head in *L*. *femoralis*, while ending before the hind margin in the other taxa.

*Limnephilus fischeri* Ruiter, 1995 has a very pale yellow head (Figure 9E) and prothoracic background color. The head and pronotum have a pattern similar to others of the *L. subcentralis* group. In the Churchill area, this group has four species (*L. fischeri, L. hageni* Banks, 1930*, L. partitus* Walker, 1852*, L. sansoni* Banks, 1918), which are most easily separated by coloration. The dark mesonotal color makes *L. fischeri* the easiest to separate from the rest. Its case is made of long, thin vegetal pieces that appear almost spiraled as in *Mystacides* or *Phryganea*. The venter of the 1^st^ abdominal segment has very few setae.

*Limnephilus hageni* larvae are very similar to the rest of the *L*. *subcentralis* group larvae (see discussion under *L. fischeri*). The indistinct postgenal band (Figure 9F) and pale pronotal base color separate *L. hageni* from the other *L. subcentralis* group larvae. The cases in the group are also very similar, comprised of long vegetal pieces. In the *L. hageni* larvae we have, the vegetal pieces are wider than those of *L*. *fischeri* although this probably has no diagnostic significance.

*Limnephilus infernalis* (Banks, 1914) has a head and pronotal color pattern (Figure 9G) similar to *L*. *externus* and *L*. *femoralis*. However, *L*. *infernalis* has accessory setae on the meso- and metafemur. The dorsoposterior half of the pronotum lacks obvious muscle scars in *L*. *infernalis*, while these dark muscle scars are obvious in both *L*. *externus* and *L*. *femoralis*.

*Limnephilus major,* like the other *L. incisus* group larva of the Churchill area (see *L. ademus*discussion above), has a solid brown background color to the head with darker muscle scars and only pale frontoclypeal markings (Figure 9H). It lacks dorsal chloride epithelia. However, it possesses accessory setae on the lateral surfaces of the meso- and metafemur. The case is made of sand grains and seems quite fragile, and readily crushed. On one specimen we looked at the pale frontoclypeal areas were extremely faint.

*Limnephilus nigriceps* (Zetterstedt, 1840) has a head and thoracic color pattern very similar to the three-banded head with wide anterior dark pronotal band. However, the head bands are nearly coalesced in most specimens to the point the head appears to have a three-spot pattern (Figure 9I). *L. nigriceps*, along with *L. infernalis*, differ from the other three-banded head taxa by possessing accessory setae on the meso- and metafemur lateral surfaces. The larvae of *L. indivisus* and *L. rhombicus* (Linnaeus, 1758) also occur in the Churchill area and are expected to have similar coloration and setation as *L. nigriceps* and *L. infernalis*[21,36,44]. *Limnephilus nigriceps* lacks dorsal chloride epithelia. In the Churchill area the case is usually made of small, thin bark pieces haphazardly arranged into a slightly triangular cross section.

*Limnephilus partitus* (Figure 9J) is very similar to *L*. *hageni* and *L. sansoni* Banks, 1918. In *L. partitus*, the muscle scars of the pronotal dorsoposterior area are widely scattered and distinct and there are few setae on the first abdominal segment.

*Limnephilus perpusillus* is another taxa with a plain brown head with darker muscle scars and very little color pattern (Figure 9K), and no accessory setae on the meso- and metafemur lateral surfaces. There is a small white triangle in the posterior apex of the frontoclypeal suture and two poorly developed anterior pale areas originating at the anterior frontoclypeal constriction and extending along the frontoclypeal suture nearly to the labrum. These anterior pale areas are located primarily laterad of the frontoclypeal suture. This color pattern is very similar to that of *L. major* (see discussion above) and *L. picturatus*. The ventral apotome of *L. perpusillus* is long (unlike *L. picturatus*) nearly extending to the posterior head margin. The case is made of dark vegetal fragments and is very smooth and round with little taper or curve.

*Limnephilus picturatus* has a yellow/brown head with darker muscle scars and a small pale band following the frontoclypeal suture from the apex to the frontoclypeal constriction (Figure 9L). The ventral apotome is short, barely half the length of the ventral ecdysial suture. The meso- and metafemur lack setae on their lateral surfaces. The case is made of fairly large vegetal pieces.

*Limnephilus sansoni* is another one of the *L. subcentralis* group (see *L. fischeri*discussion above) with the pale yellow head and typical medial dark band on the frontoclypeus (Figure 10A); dark U-shaped band outside the frontoclypeal suture; and dark band in the transverse pronotal depression. The thoracic setae are greatly reduced in number with the thoracic sa1 and sa2 reduced to one or two large setae, often just one. The two major setae of the forefemur ventral margin are not both pale, with the black located distally. The case is similar to others of the group, comprised of long vegetal pieces.

*Limnephilus sericeus* larvae have a dark brown head with faint, darker muscle scars (see *L. argenteus*discussion above). There are three obvious pale spots on the frontoclypeus (Figure 10B). These anterior pale areas are located primarily outside the frontoclypeal sutures and do not extend anteriorly much past the eye. This color pattern is similar to *L. ademus*, from which it can be distinguished by the absence of meso- and metafemur accessory setae. The case is made of vegetal parts arranged in a smooth cylinder. The larva was described by Lepneva [17].

##### Nemotaulius

A single *Nemotaulius hostilis* larval specimen was collected at Churchill. This species can be readily separated from other Churchill caddis larvae by the combination of: distinct head and pronotal coloration similar to the *L. subcentralis* group with the medial frontoclypeal dark band surrounded by the U-shaped dark band located laterad of the frontoclypeal sutures (Figure 10C); the pronotum with a dark, transverse band about midlength; meso- and metafemur accessory setae present. While the case is usually flattened in immature specimens (see Wiggins [21]), the mature case is often round but still made of fairly large vegetal pieces.

##### Onocosmoecus

*Onocosmoecus unicolor* is the only dicosmoecin collected so far in Churchill and its larva has not been associated in this study. The numerous large, pale setae along the mesofemur ventral margin, along with the medial stripe of the meso- and metathorax readily separate this taxa from all other Churchill limnephilids. We have included a photo of a specimen associated from Montana where it is fairly common (Figure 10D). The case is usually made of small vegetal pieces arranged into a very smooth, slightly curved and tapered case [21]. The larvae were described in Wiggins & Richardson [45].

##### Phanocelia

*Phanocelia canadensis* (Figure 10E) is the only limnephilid collected so far in the Churchill area with gill clusters of a single filament. Wiggins [21] provided a description. The larvae are small, about 10 mm, and the case is poorly constructed, tending to fall apart.

##### Philarctus

Wiggins [21] pointed out the difficulty of separating *Philarctus bergrothi* McLachlan, 1880 larvae [as *P. quaeris* (Milne, 1935)] from those of *Asynarchus*, *Clistoronia*, and some *Limnephilus* based on head pattern (Figure 10F). It can be separated from *Clistoronia* (which has not been collected in the Churchill area) by the presence of accessory setae on the mesofemur lateral surfaces (lacking in *Clistoronia*). *Philarctus* can be separated from *Anabolia* and *Asynarchus*, which the 3-spot head pattern resembles, by the lack of dorsal chloride epithelia (present in *Anabolia* and *Asynarchus*). It can be separated from those *Limnephilus* with mesofemur accessory setae by the presence of accessory setae on the basal mesotrochanter segment in *Philarctus*. The case is often constructed of mollusks as figured by Wiggins [21], although immature specimens may use small vegetation pieces, switching to mineral or shells prior to pupation.

#### Molannidae

**Figure 11:**
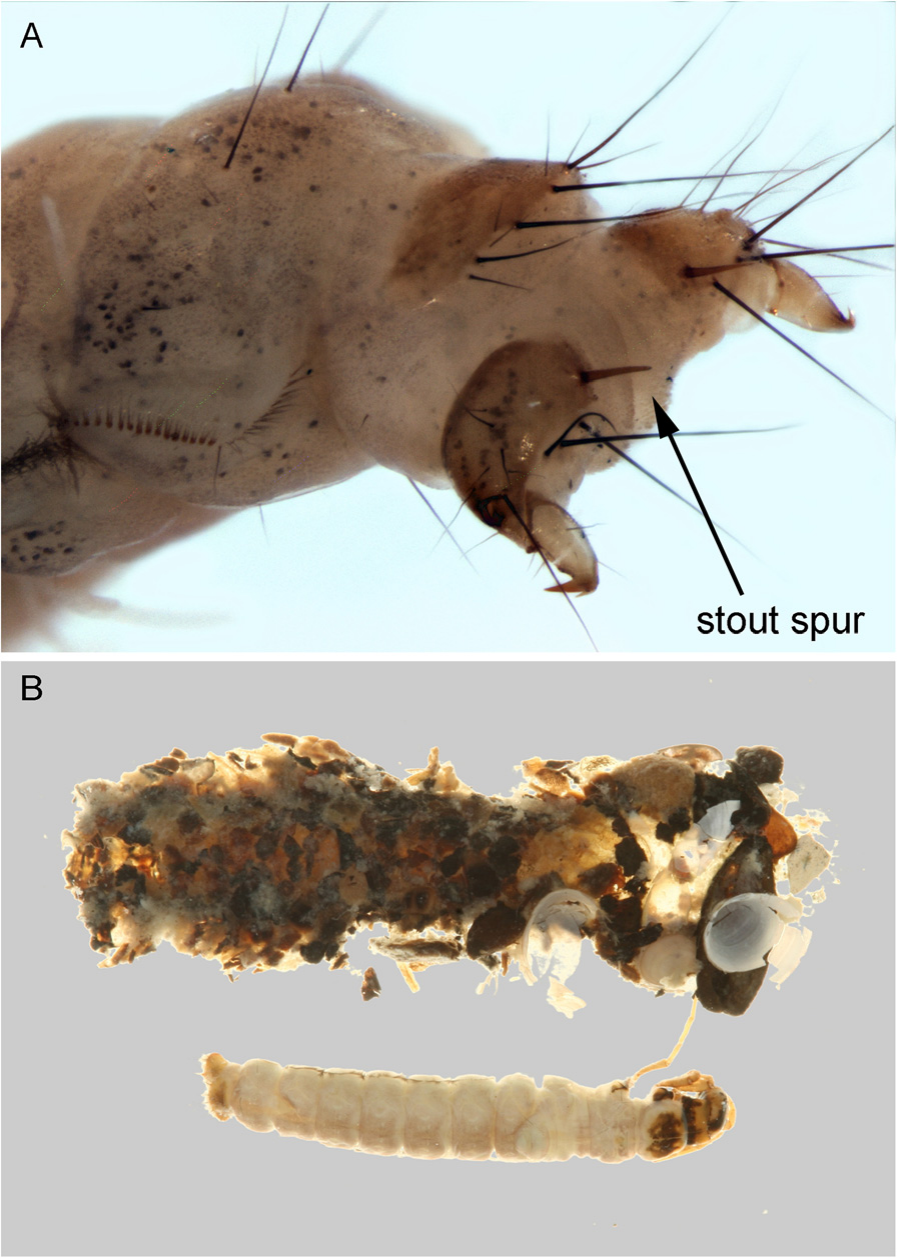
***Molanna flavicornis*: A: apex of abdomen, oblique; B: habitus, dorsal.**

Only a single molannid species, *Molanna flavicornis* Banks, 1914, has been collected in the Churchill area. Wiggins [21] provides diagnostic information for this genus and *M*. *flavicornis* is the only known molannid with a stout spur at the base of the anal proleg (Figure 11). This is another widespread northern taxon with occurrence reported from most of Canada, the northern tier of states and extending down the Rockies as far as Colorado.

#### Philopotamidae

*Chimarra socia* Hagen, 1861, the only philopotamid species collected so far in the Churchill area, can be easily separated from the other Churchill caddis at the family level. Larvae have yet to be collected in the Churchill area.

#### Phryganeidae

Larvae for 7 of the 11 Churchill area phryganeids have been associated via COI. The larvae of 3 of the genera [*Banksiola crotchi* Banks, 1943, *Phryganea cinerea* Walker, 1852, and *Ptilostomis semifasciata* (Say, 1828)] can be readily separated at the genus level (see Wiggins [21,46]) from the more diverse Churchill *Agrypnia*.

##### Agrypnia

**Figure 12:**
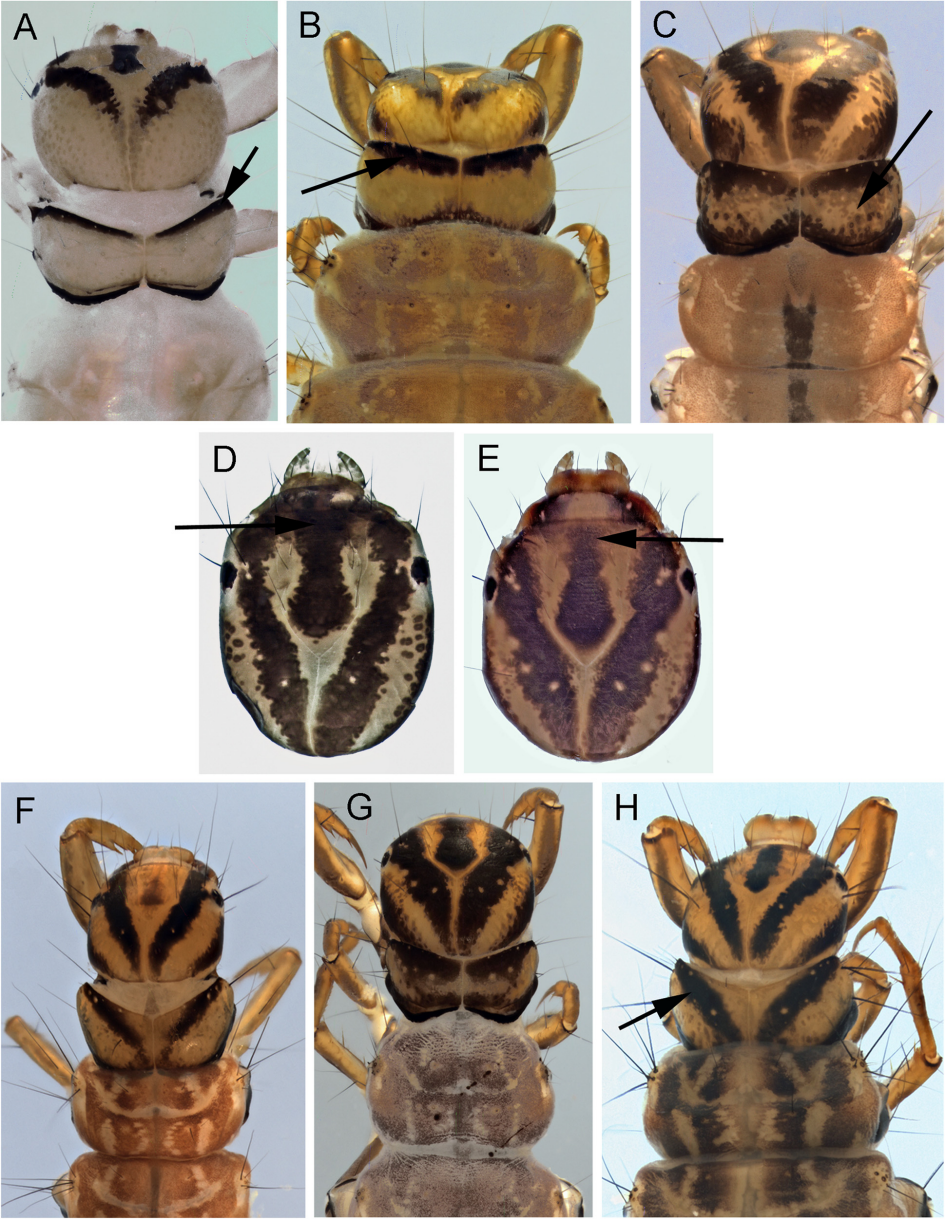
***Agrypnia colorata *A: dorsal; *Agrypnia deflata *B: dorsal; *Agrypnia glacialis *C & D: dorsal; *Agrypnia improba*, F: dorsal. *Agrypnia pagetana *E & G: dorsal, *Agrypnia straminea *H: dorsal.**

*Agrypnia* is the second most diverse caddisfly genus (after *Limnephilus*) in the Churchill area. Eight of the ten species reported from North America [46] have been collected in the Churchill area and we associated six larvae via COI. While Wiggins [46] indicated the difficulty of using the distinctive head and thorax color to separate the phryganeid genera, the color patterns of the six known Churchill larvae proved useful for separating the *Agrypnia* species. Comparative pictures of the associated Churchill area larvae are presented in Figure 12.

*Agrypnia colorata* Hagen, 1873 larvae [along with *A. deflata* (Milne, 1931)*, A. glacialis* Hagen, 1873*,* and *A. pagetana* Curtis, 1835] belong to a North American group with a dark, transverse band at the anterior and posterior margins of the pronotum (Figure 12A). *A. colorata* can be separated from the other three by the narrow anterior dark band *vs.* the broad and/or blotchy anterior pronotal bands in the other banded taxa. The two *A. colorata* larvae examined exhibited color variation on the lateral side of the head that are not evident in the other species. One of the specimens lacked the dark speckling laterally and the dark blotch ventrally. This color variation is likely the result of differences in the age of the specimens and points out the need to use mature specimens for physical characters to separate species.

*Agrypnia deflata* (Figure 12B) can be separated from the other larvae with banded pronotum by the fairly wide anterior band that lacks the additional dark spotted areas between the anterior and posterior pronotal bands.

*Agrypnia glacialis*, along with *A. pagetana*, have the anterior pronotal transverse dark band consisting more of a blotch with the anterior band extending towards the posterior band as an area of merged dark spots and blotches (Figure 12C). Only a single DNA associated *A. glacialis* was available for study and it is immature, perhaps a 4^th^ instar based on size. However it clearly exhibits the key hole shaped mesal frontoclypeal band (Figure 12D) of its Eurasian sister species *A. picta* Kolenati, 1848 (see Wallace et al. [39], Figure 104E). The frontoclypeal medial band does not reach the posterior margin of the frontoclypeus as in *A. pagetana*.

*Agrypnia pagetana* can be separated from the other known Churchill larvae with transverse bands by the combination of the blotched, dark, anterior pronotal band and the dark mesal frontoclypeal band not expanding at its anterior end (Figure 12E&G). These characters were also described by Wallace et al. [39]. Unlike *A. glacialis*, the posterior end of the frontoclypeal medial band in *A. pagetana* reaches the posterior margin of the frontoclypeus. Only a single associated specimen of *A. pagetana* is available for study.

The anterior pronotum of *A. improba* (Hagen, 1873) and *A. straminea* Hagen, 1873 are part of the group which have two diagonal anterior pronotal bands directed posteromesally. These bands appear as a “V” with the apex of the “V” at the posteromesal pronotal margin in dorsal view (Figures 12F&H).

*Agrypnia improba* has a relatively pale, incomplete, medial frontoclypeal band (Figure 12F). This character separates it from *A. straminea* and Wiggins [46] provides an excellent figure for *A. improba*.

*Agrypnia straminea* had a distinct, wide, dark medial band on the frontoclypeus (Figure 12H), which separates it from *A. improba. A. straminea* is also figured by Wiggins [46].

##### 

**Banksiola** A single larva of *Banksiola crotchi* is associated from the Churchill area. It matches the description and figures provided by Wiggins [46].

##### 

**Phryganea** Larvae for *Phryganea cinerea* have not been collected during this study. Wiggins [46] provided a description and figure of this species but noted that he could not separate *P. cinerea* from the other associated *Phryganea* he had available.

##### 

**Ptilostomis** Larvae for *Ptilostomis semifasciata* have not been collected during this study. Wiggins [46] provided a description and figure of *P. semifasciata*. But, like in *Phryganea*, he noted that he could not separate *P. semifasciata* from the other associated *Ptilostomis* he had available.

#### Polycentropodidae

**Figure 13:**
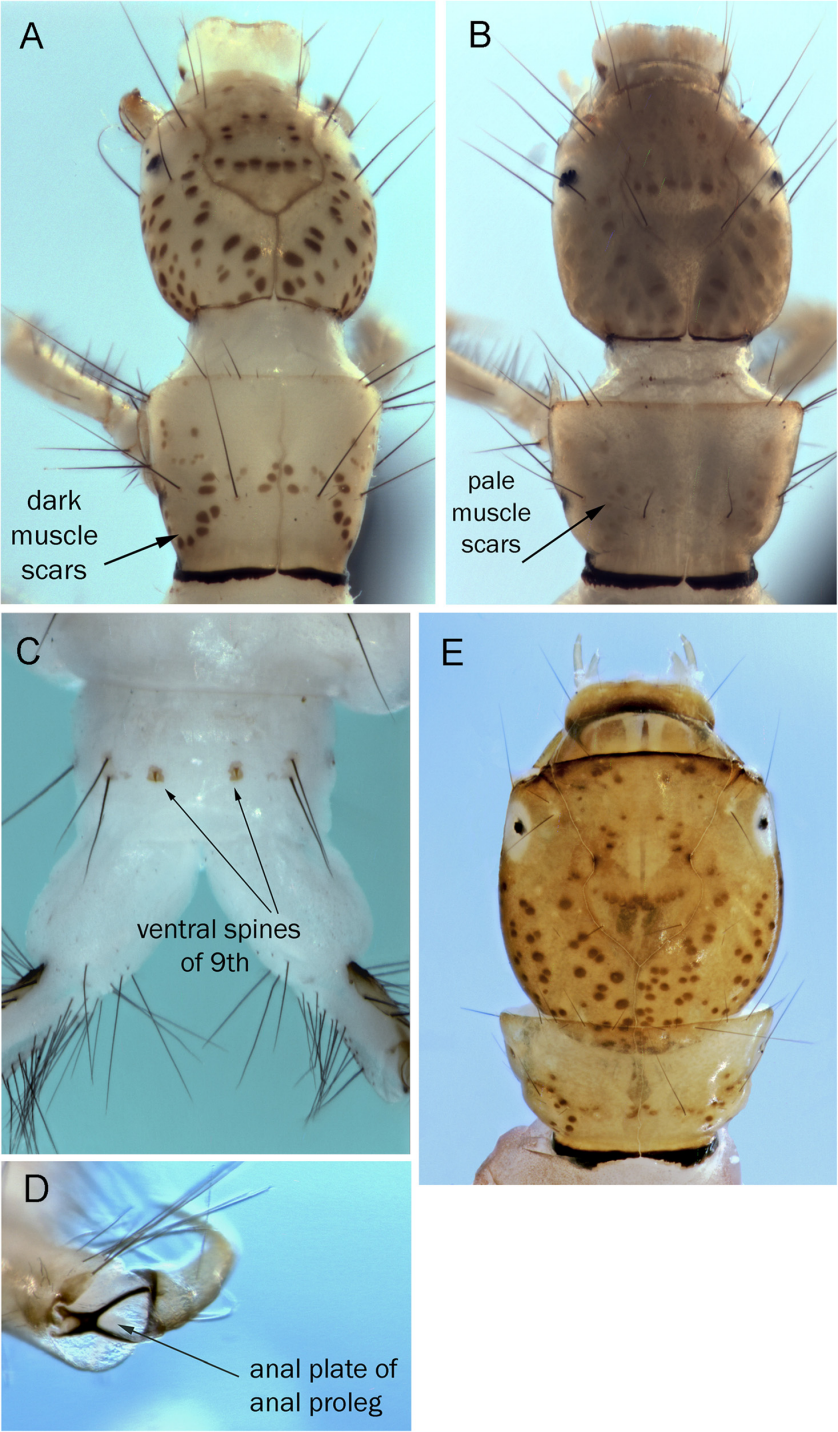
***Neureclipsis crepuscularis *A: head, dorsal; *Neureclipsis valida *B: head, dorsal, C: 9th sternite; *Polycentropus aureolus *D: anal proleg, dorsal, E: head, dorsal.**

Larvae for four Churchill polycentropids have been associated via DNA barcoding. *Polycentropus* are readily separated from *Neureclipsis* by the presence of two dark sclerotized bands on the dorsal plate of the anal proleg (Figure 13D), in addition to other characters provided by Wiggins [21].

*Neureclipsis crepuscularis* (Walker, 1852) has numerous dark muscle scars on the prothorax (Figure 13A), which separates it from *N. valida* (Figure 13B). Wiggins [21] indicated that some *Neureclipsis* have a pair of short stout setae on the venter of segment nine. These stout setae appear to be a reduction in size of two long setae at this position and are absent in *N. crepuscularis*.

*Neureclipsis valida* has faint muscle scars on the pronotum (Figure 13B). The stout 9^th^ sternal ventral setae of *N. bimaculatus* (see Wiggins [21]) are also present in *N. valida* (Figure 13C).

*Polycentropus aureolus* (Banks, 1930) could not be separated from *Polycentropus smithae* Denning, 1949 based on head coloration. They both have heads with a pale background covered with scattered dark muscle scars (Figure 13E).

#### Psychomyiidae

The larvae of the single psychomiid (*Psychomyia flavida* Hagen, 1861) have not been collected in the Churchill area. However, both Ross [47] and Flint [48] provided descriptions. It can be readily separated from the other Churchill caddisflies by the presence of the large submental sclerites and the hatchet-shaped trochantin (see Wiggins [21]).

#### Rhyacophilidae

**Figure 14:**
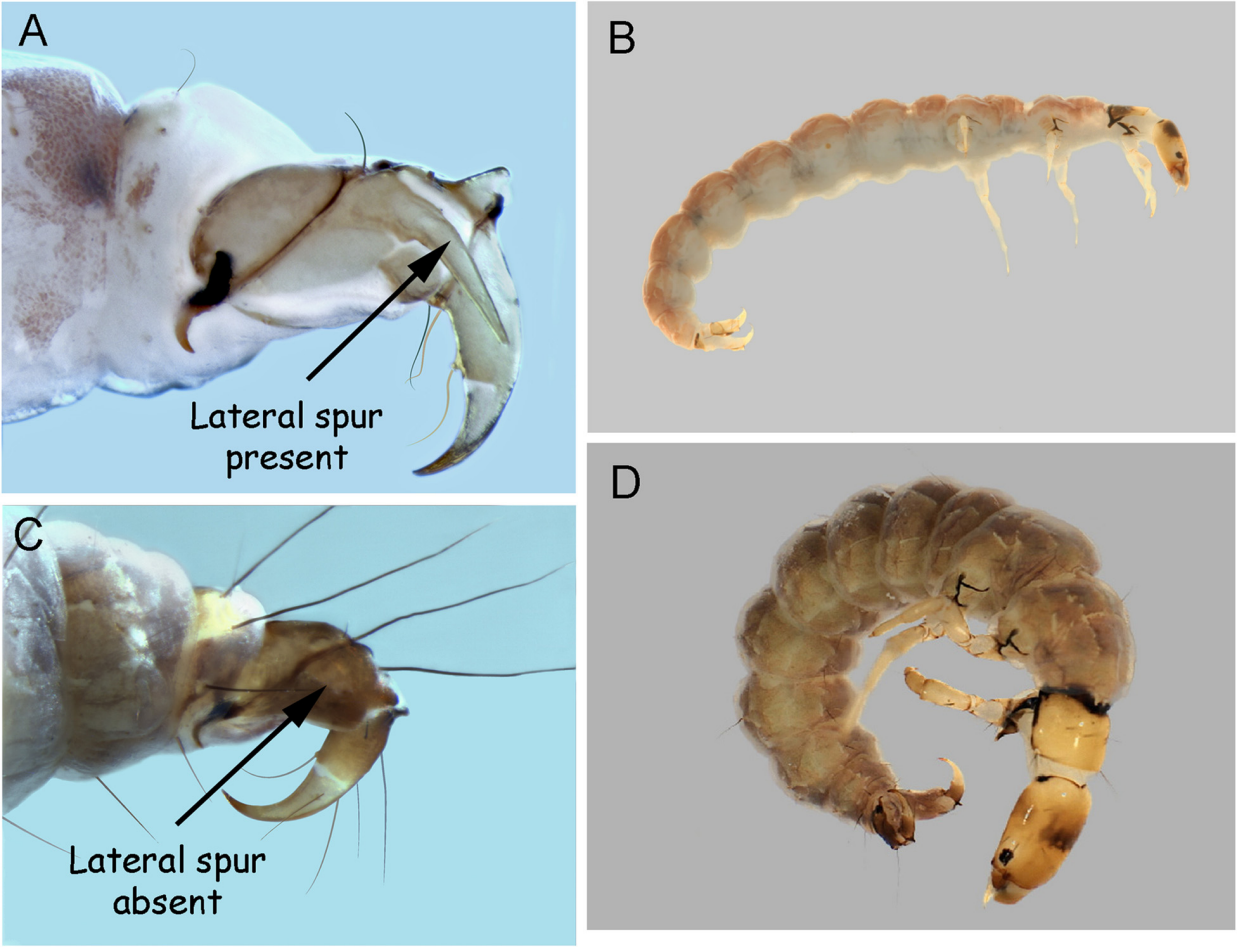
***Rhyacophila angelita *A: anal proleg, lateral; B: habitus, lateral; *Rhyacophila mongolica *C: anal proleg, lateral; D: habitus, lateral.**

Larvae of both the Churchill area *Rhyacophila* (*R. angelita* Banks, 1911 and *R*. *mongolica*) were associated. They can be easily separated by the presence of a curved spike on the lateral sclerite on the anal proleg in *R*. *angelita* (Figure 14). The larva of *R*. *mongolica* is tentatively associated via DNA to adult specimens of the species collected in Mongolia. Wiggins and Parker [49] reported *R. mongolica* adults from the Yukon.

##### Larval key for Limnephilidae

The following key (Table 2) was developed from DNA associated material from the Churchill area. It was only developed for the Limnephilidae as the rest of the fauna was represented by relatively few species, which could be readily separated in the text or at the generic level. It must be recognized that this key may not work as well in other parts of the world. This is particularly true at the species level. While some of the Churchill species were very abundant, larvae of others were rarely collected and the characters below may be based on only a specimen or two.

**Table 2:** Key for the Churchill, Manitoba, area Limnephilidae larvae

1)	all gill clusters with a single filament	*Phanocelia canadensis*
		
	at least some gills with clusters of 3 or more filaments (Figure 15A)	2
		
2)	at least some gill clusters with more than 3 filaments (Figure 15A)	3
		
	no gill clusters with more than 3 filaments (Figure 15D)	5
		
3)	setae present on metonotal membrane between sa2 sclerites (Figure 15B)	*Hesperophylax designatus*
		
	metanotal membrane setae absent	4
		
4)	2nd and 3rd mesofemur with only 2 major ventral setae (Figure 15C)	*Lenarchus fautini*
		
	2nd and 3rd mesofemur with numerous major ventral setae (Figure 15F)	*Onocosmoecus unicolor*
		
5)	setae present on metonotal membrane between sa2 sclerites (Figure 15B)	*Asynarchus rossi*
		
	metanotal membrane setae absent	6
		
6)	dorsomesal chloride epithelia present (Figure 15D)	7
		
	dorsomesal chloride epithelia absent	12
		
7)	mesofemur with accessory setae on at least one lateral surface (Figure 15C)	8
		
	mesofemur lacking lateral accessary setae	11
		
8)	anterolateral corner of pronotum with scurf of small, stout spicules (Figure 15E)	*Anabolia bimaculata*
		
	stout spicule scurf absent	9
		
9)	basal trochantal segment with accessory setae (Figure 16A & B)	10
		
	basal trochantal segment without accessory setae	*Asynarchus mutatus*
		
10)	ventrolateral abdominal gill series extends to 4th segment	*Asynarchus lapponicus*
		
	ventrolateral abdominal gills extend to 6-7th segment	*Asynarchus montanus*
		
11)	ventral apotome short, about half the length of ventral ecdysial suture (Figure 16C); metanotal sa1 and sa2 sclerites small or absent	*Arctopora pulchella*
		
	ventral apotome long, nearly reaching posterior margin (Figure 16D); metanotal sclerites large, obvious (Figure16E)	*Grammotaulius interrogationis*
		
12)	accessory setae present on at least one mesofemur lateral surface (Figure 15C &16B)	13
		
	mesofemur lateral accessary setae absent	19
		
13)	basal segment of mesotrochanter with accessary setae (Figure 16B)	*Philarctus bergrothi*
		
	mesotrochanter basal segment without accessory setae	14
		
14)	dorsum of head base color pale with a distinct U-shaped dark band (Figure 10C)	*Nemotaulius hostilus*
		
	base color of head dark (Figure 9I)	15
		
15)	anterior of pronotum with transverse, wide dark band (Figure 16E)	16
		
	anterior of pronotom without wide, transverse dark band	17
		
16)	head with three distinct dark bands dorsally against pale background (Figure 9G)	*Limnephilus infernalis*
		
	head with dark areas and scattered darker muscle scars, without pale background laterally (Figure 9I)	*Limnephilus nigriceps*
		
17)	pale blotches of frontoclypeal area all located within frontoclypeal sutures (Figure 8K)	*Limnephilus alaicus*
		
	at least parts of frontoclypeal pale areas located laterad of frontoclypeal sutures (Figure 8J)	18
		
18)	the two anterior frontoclypeal pale areas are round (Figure 8J)	*Limnephilus ademus*
		
	the two anterior frontoclypeal pale areas are linear and extend further forward along frontoclypeal suture (Figure 9H)	*Limnephilus major*
		
19)	head with dark background color with darker muscle scars. Any pale areas are small and limited to the frontoclypeal area (Figure 9L)	20
		
	head with large pale areas not limited to frontoclypeal area (Figure 9A)	23
		
20)	head monochromatic brown with little evidence of pale areas in frontoclypeal area (Figure 8L)	*Limnephilus argenteus*
		
	head with pale areas in frontoclypeal area (Figure 10B)	21
		
21)	anterior ventral apotome about half the length of the ecdysial suture (Figure 16C)	*Limnephilus picturatus*
		
	anterior ventral apotome nearly as long as the ecdysial suture (Figure 16D)	22
		
22)	frontoclypeal pale blotches extend anteriorly beyond eye (Figure 9K)	*Limnephilus perpusillus*
		
	frontoclypeal pale blotches do not extend anteriorly beyond eye (Figure 10B)	*Limnephilus sericeus*
		
23)	frontoclypeus without distinct linear dark stripe (Figure 9A)	*Limnephilus canadensis*
		
	frontoclypeal stripe distinct (Figure 9D)	24
		
24)	medial frontoclypeal band expanded anteriorly with dark coloration reaching anterior frontoclypeal corners (Figure 9C)	25
		
	medial frontoclypeal band not strongly expanded and without distinct dark coloration in anterior frontoclypeal corners (Figure 9F)	27
		
25)	lateral dark bands of head reach posterior margin of head (Figure 9D)	*Limnephilus femoralis*
		
	lateral dark bands curved mesad and not reaching posterior margin of head (Figure 9B)	26
		
26)	mesonotal sa1 with numerous setae	*Limnephilus externus*
		
	mesonotal sa1 with only a single seta (Figure 16F)	*Limnephilus extractus*
		
27)	mesonotal base color dark (Figure 17A)	*Limnephilus fischeri*
		
	mesonotal base color pale (Figure 17B)	28
		
28)	dark muscle scars broadly scattered across postgenal area (Figure 17C)	*Limnephilus sansoni*
		
	postgenal muscle scars coalesced into a band	29
		
29)	postgenal band distinct (Figure 17D)	*Limnephilus partitus*
		
	postgenal band indistinct (Figure 17E)	*Limnephilus hageni*

**Figure 15:**
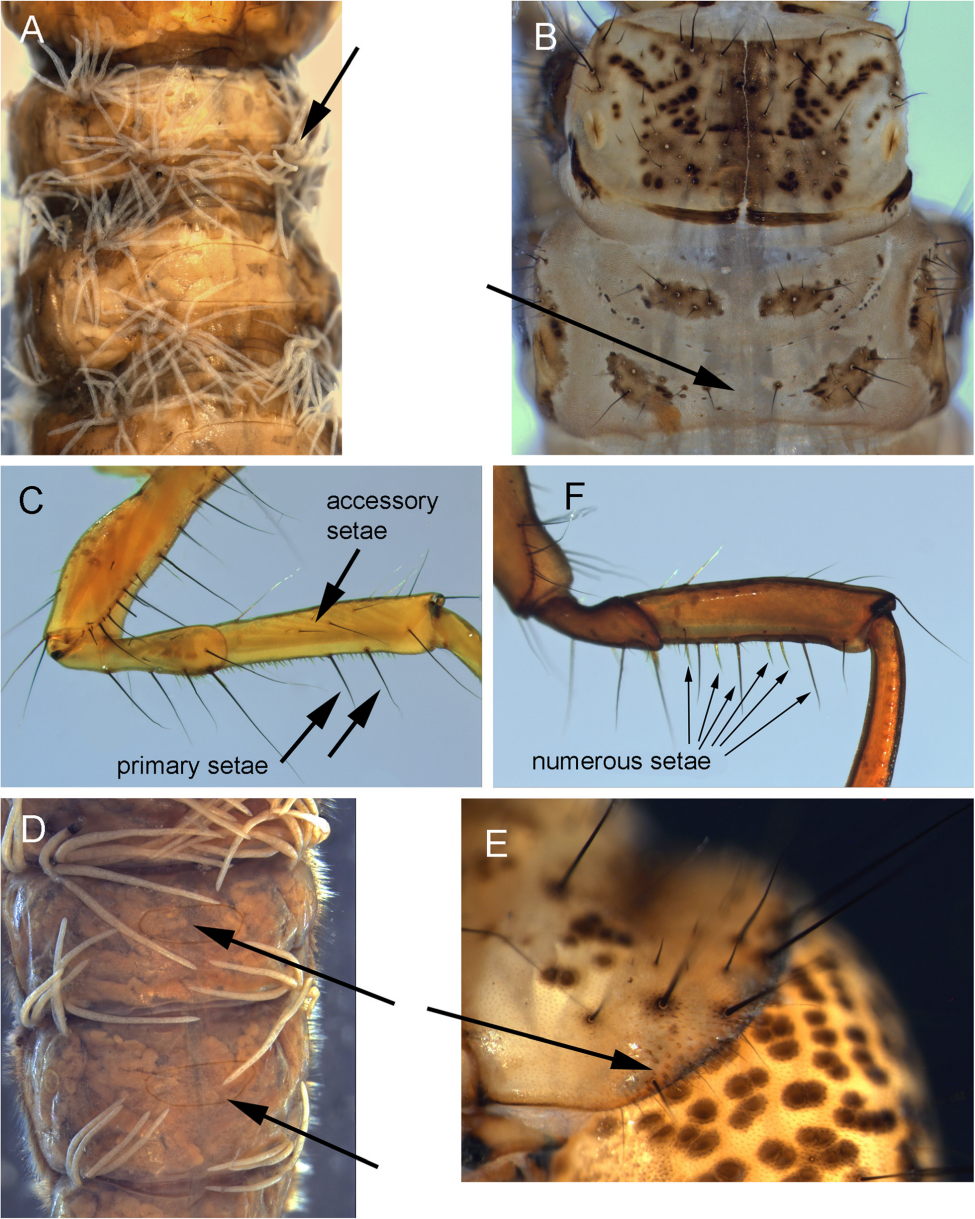
***Lenarchus *sp. A: abdomen, ventral; *Asynarchus rossi*; B: thorax, dorsal; *Anabolia bimaculata *C: 2nd leg, posterior view; D: abdomen, dorsal; E: prothorax, lateral; *Onocosmoecus unicolor, *F: 2nd leg, posterior.**

**Figure 16:**
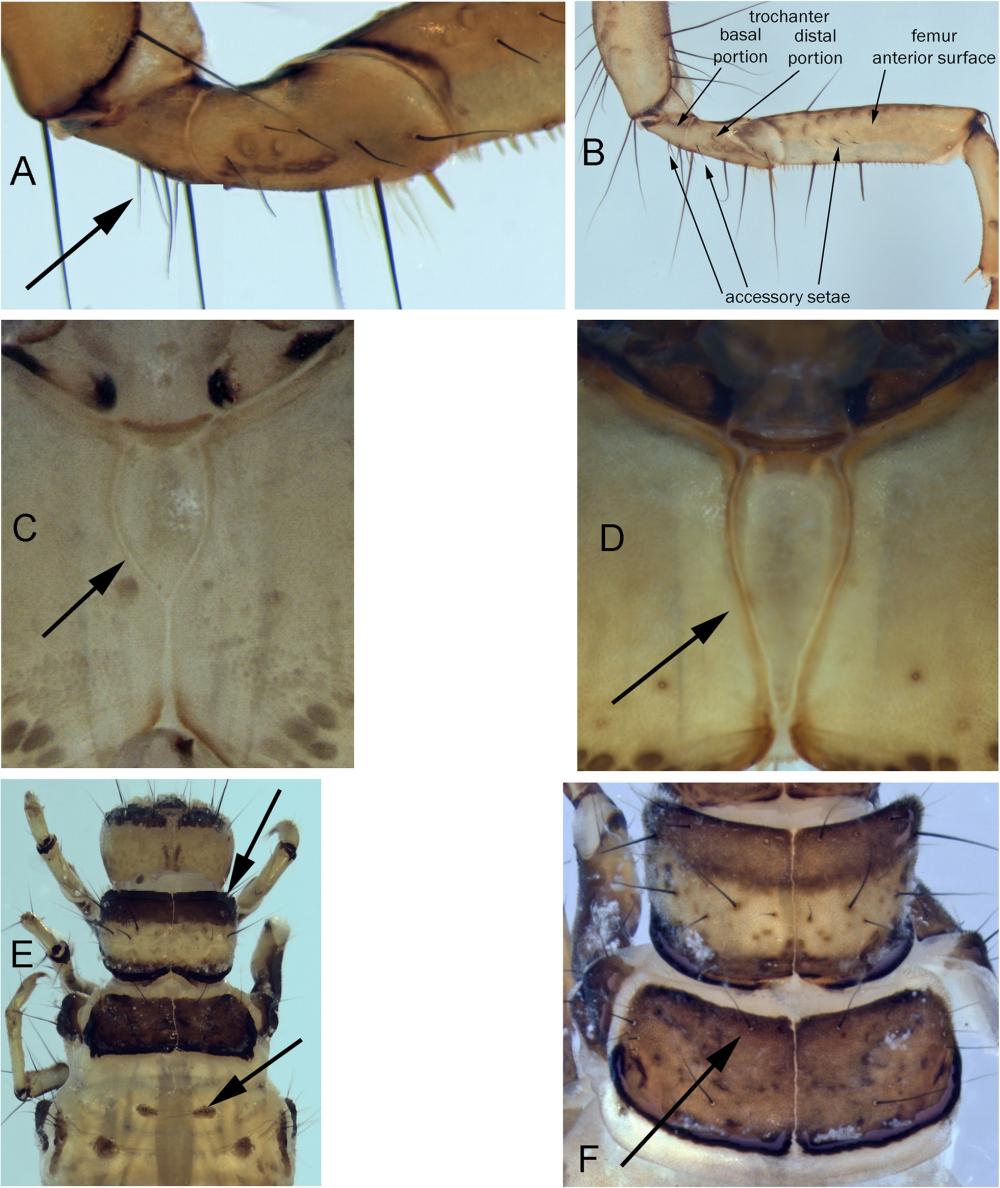
***Asynarchus lapponicus *A: mesotrochanter, posterior view; *Philarctus bergrothi *B: 2nd leg, anterior view; *Arctopora pulchella *C: ventral apotome; *Grammotaulius interrogationis *D: ventral apotome; *Limnephilus infernalis *E: thorax, dorsal; *Limnephilus extractus *F: thorax, dorsal.**

**Figure 17:**
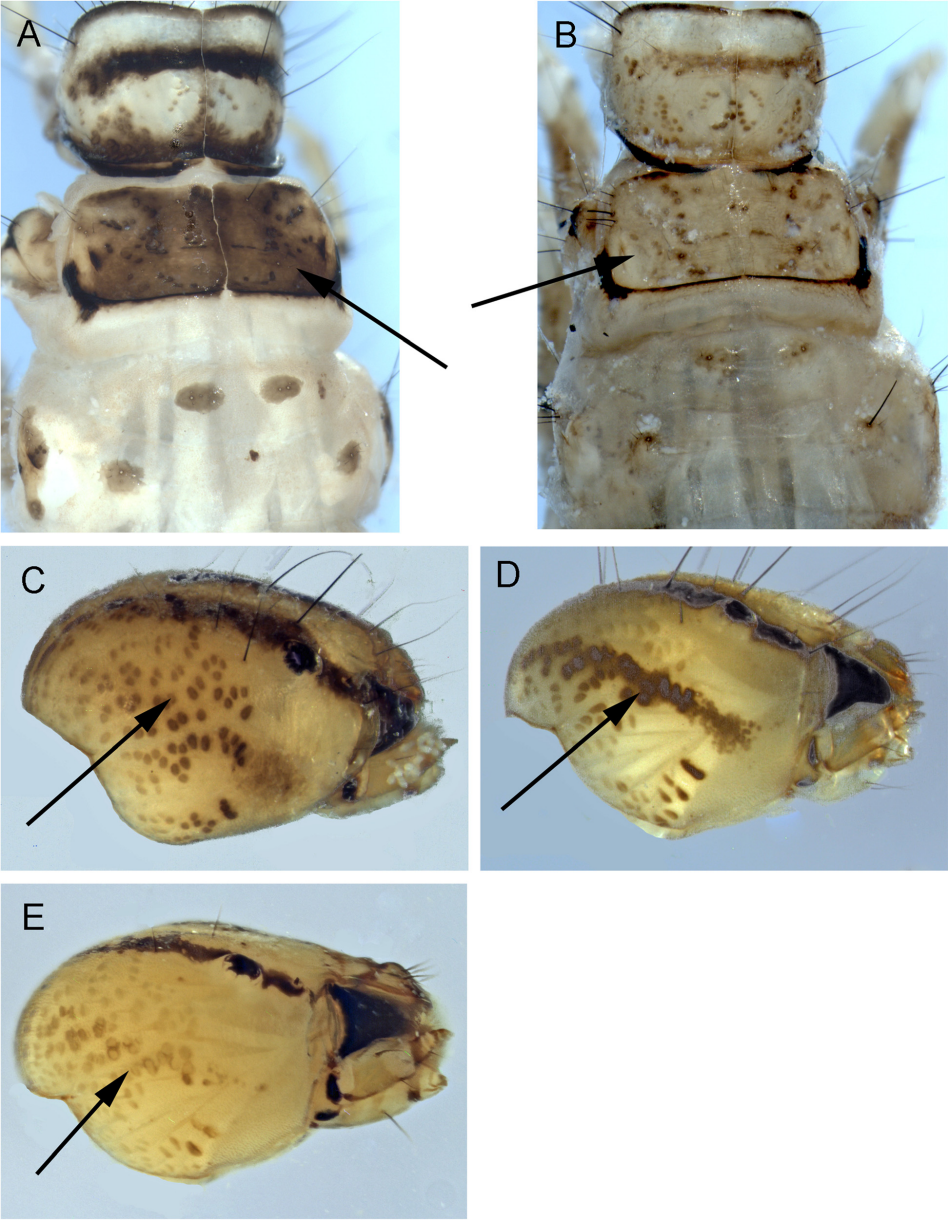
***Limnephilus fischeri *A: thorax dorsal; *Limnephilus sansoni *B: thorax, dorsal, C: head, lateral; *Limnephilus partitus *D: head, lateral; *Limnephilus hageni *E: head, lateral.**

Most of the characters used below rely on the use of mature larvae, 5th instar, or perhaps 4th. As many characters are based on color patterns, which have proven very consistent in the examined material, these colors will not be as clear in teneral specimens. One other character that has proven very useful is the presence or absence of abdominal dorsal chloride epithelia, which, however, are often difficult to see clearly. Wiggins [21] provided the best approach to observe these characters, i.e., by varying the direction and intensity of the illumination. Lateral illumination often works best. If the chloride epithelia are present, the sclerotized ring surrounding the chloride epithelia can always be found. It is useful to look at the ventral surface before looking at the dorsal surface. That will provide a better estimate of the expected coloration although it seems the dorsal chloride epithelia are always fainter than the ventral.

## Discussion

**Figure 18:**
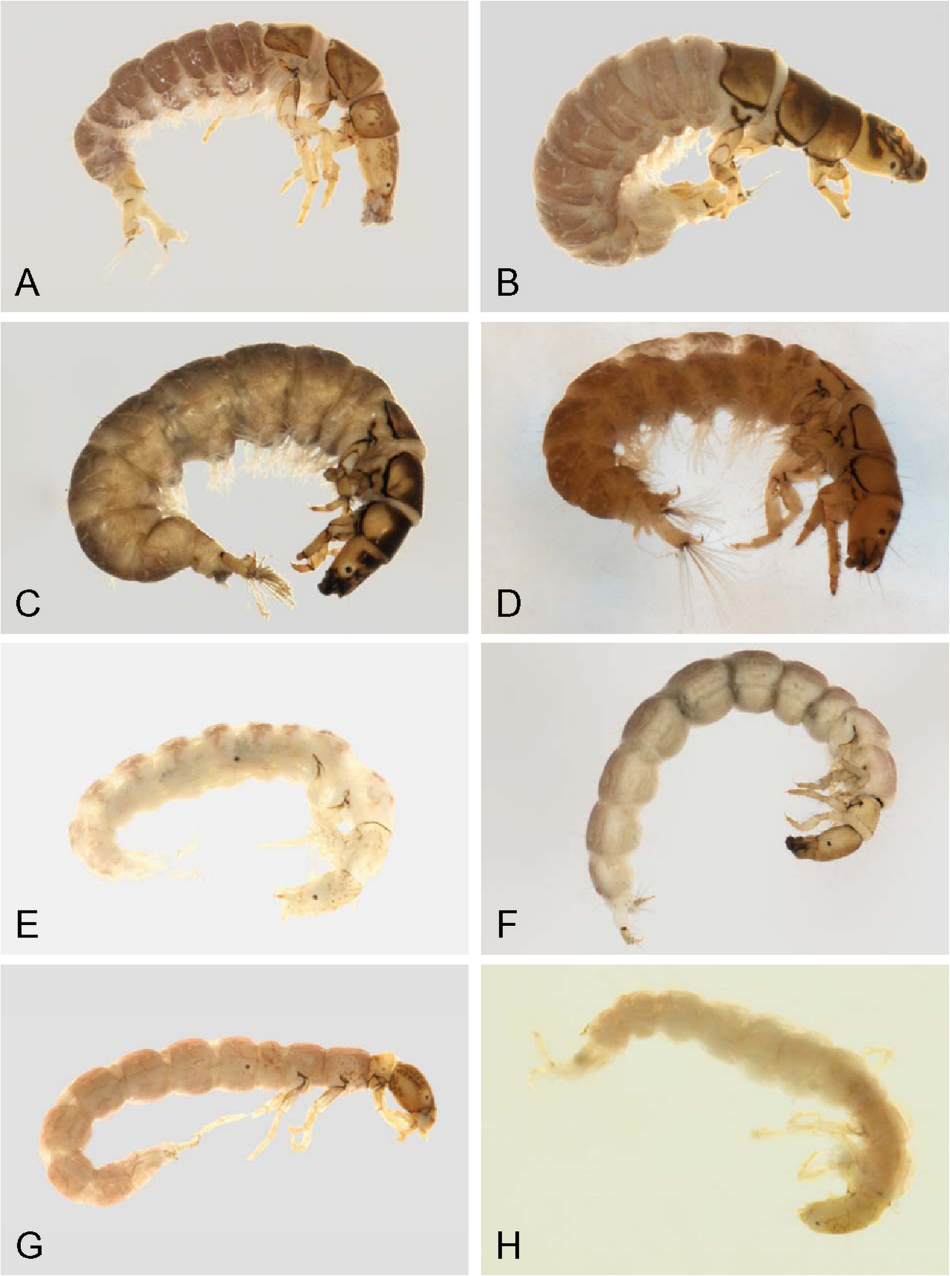
**Hydropsychidae, lateral view A: *Arctopsyche ladogensis, *B: *Hydropsyche alternans, *C: *Hydropsyche bronta, *D: *Hydropsyche vexa*; Polycentropodidae, lateral view E: *Neureclipsis crepuscularis, *F: *Neureclipsis valida *G: *Polycentropus aureoles, *H: *Polycentropus smithae.***

**Figure 19:**
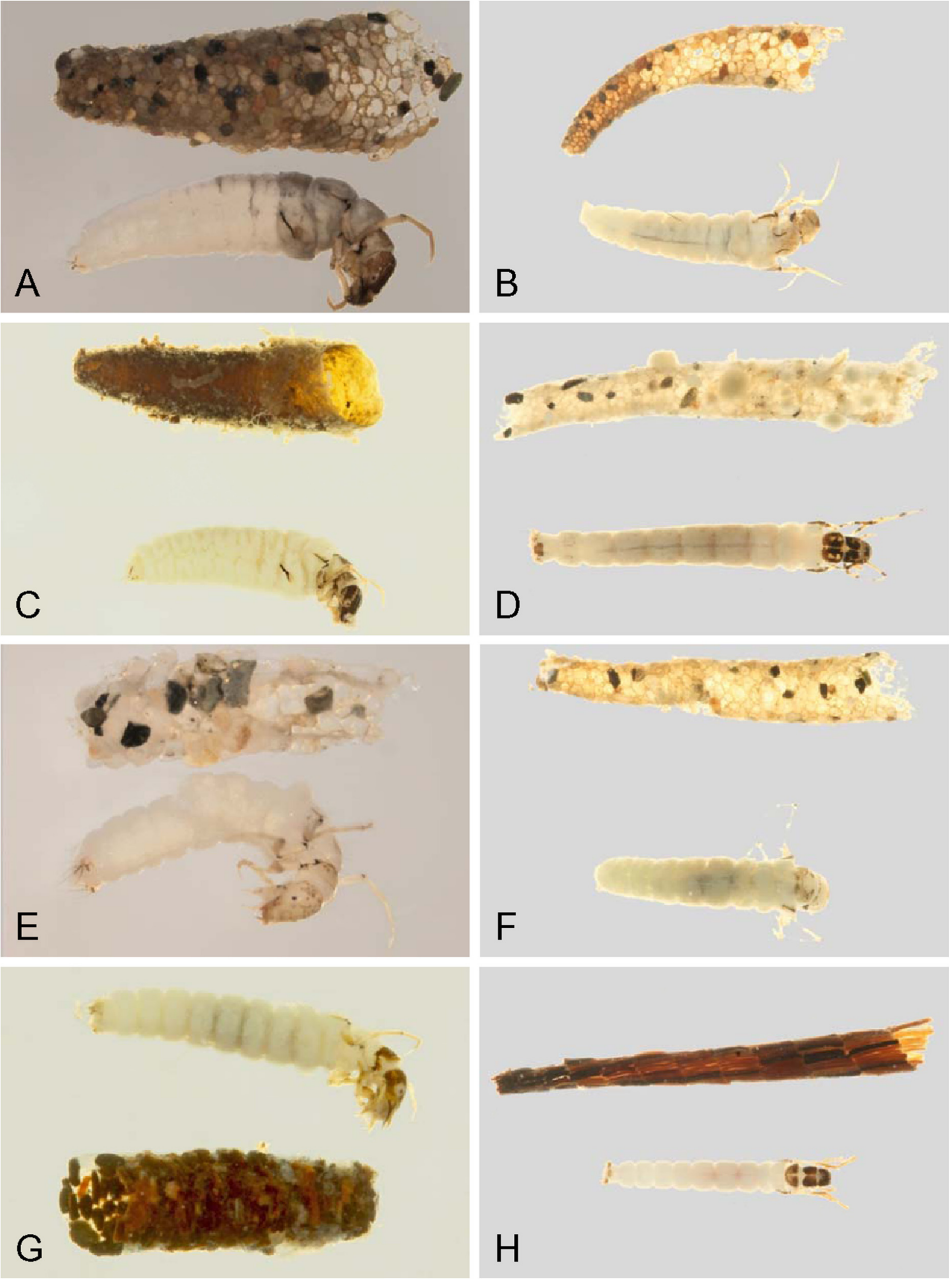
**Leptoceridae A: *Ceraclea annulicornis, *lateral; B: *Ceraclea excisa, *dorsal; C: *Ceraclea nigronervosa, *lateral; D: *Mystacides interjecta, *dorsal; E: *Oecetis *cf. *ochracea *CHU1, lateral; F: *Oecetis immobilis, *dorsal; G: *Oecetis ochracea, *lateral; H: *Triaenodes frontalis, *dorsal.**

**Figure 20:**
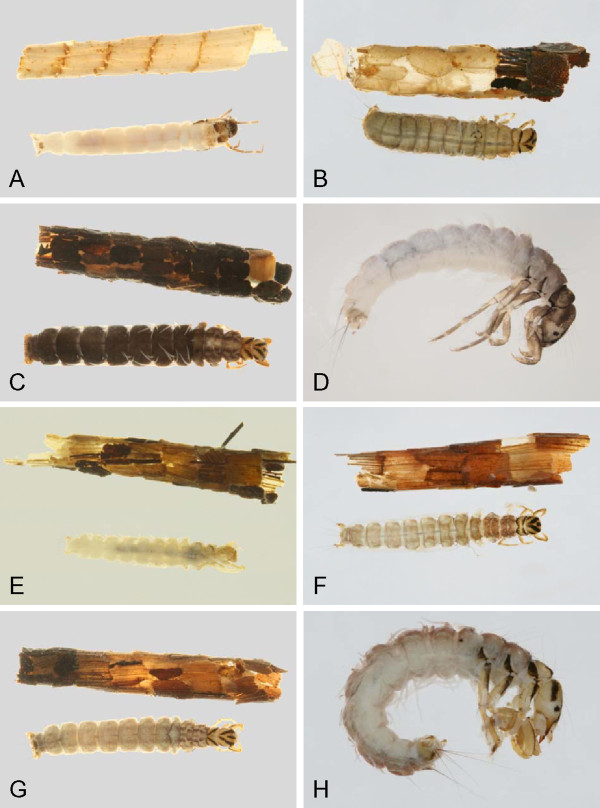
**Leptoceridae A: *Triaenodes reuteri*, lateral; Phryganeidae B: *Agrypnia colorata, *dorsal; C: *Agrypnia deflata, *dorsal; D: *Agrypnia glacialis, *lateral; E: *Agrypnia improba, *ventral; F: *Agrypnia pagetana, *dorsal; G: *Agrypnia straminea, *dorsal; H: *Banksiola crotchi, *lateral.**

**Figure 21:**
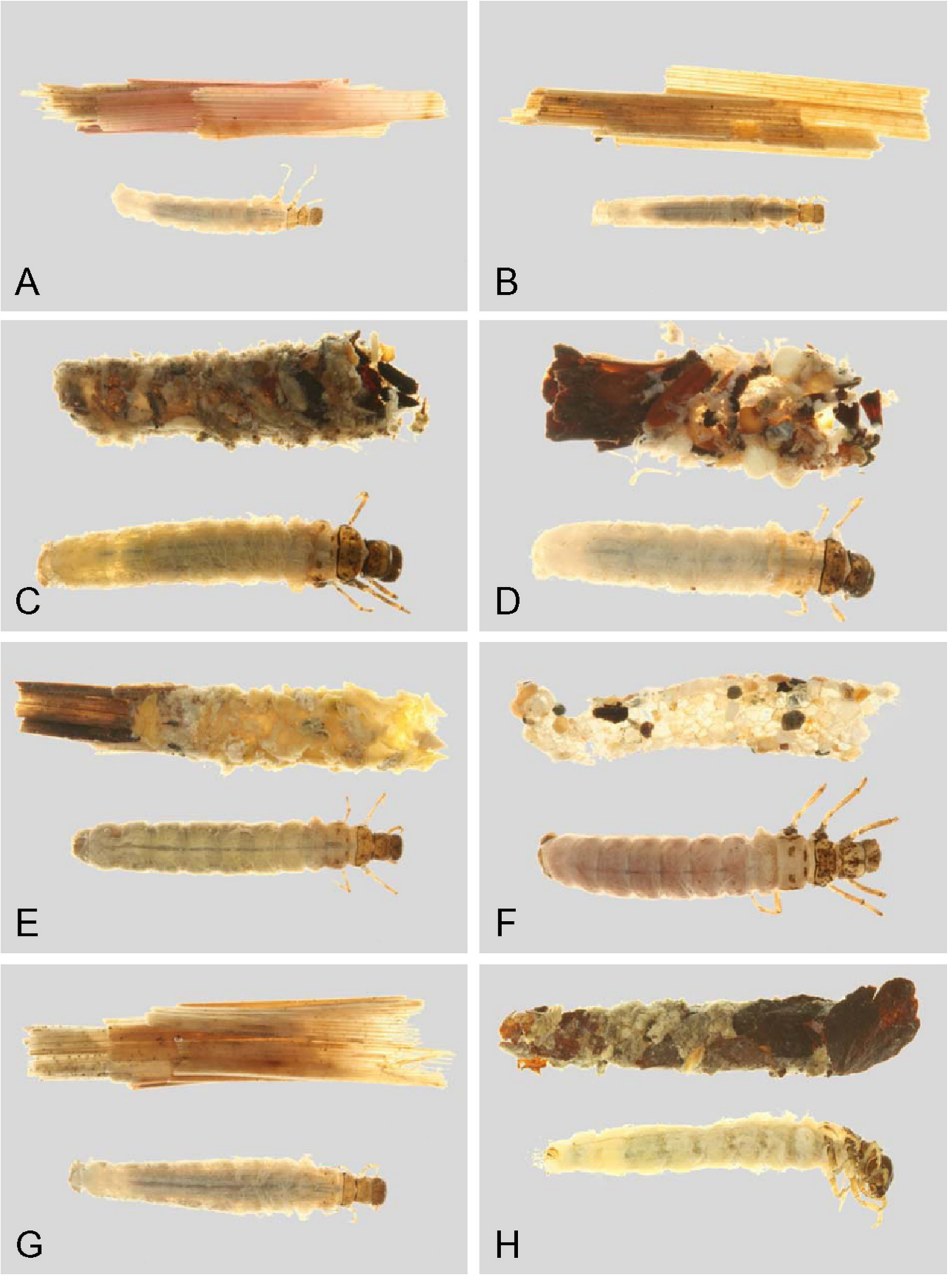
**Limnephilidae A: *Anabolia bimaculata*, dorsal; B: *Arctopora pulchella, *dorsal; C: *Asynarchus lapponicus, *dorsal; D: *Asynarchus montanus, *dorsal; E: *Asynarchus mutatus, *dorsal; F: *Asynarchus rossi, *dorsal; G: *Grammotaulius interrogationis, *dorsal; H: *Lenarchus fautini, *lateral.**

**Figure 22:**
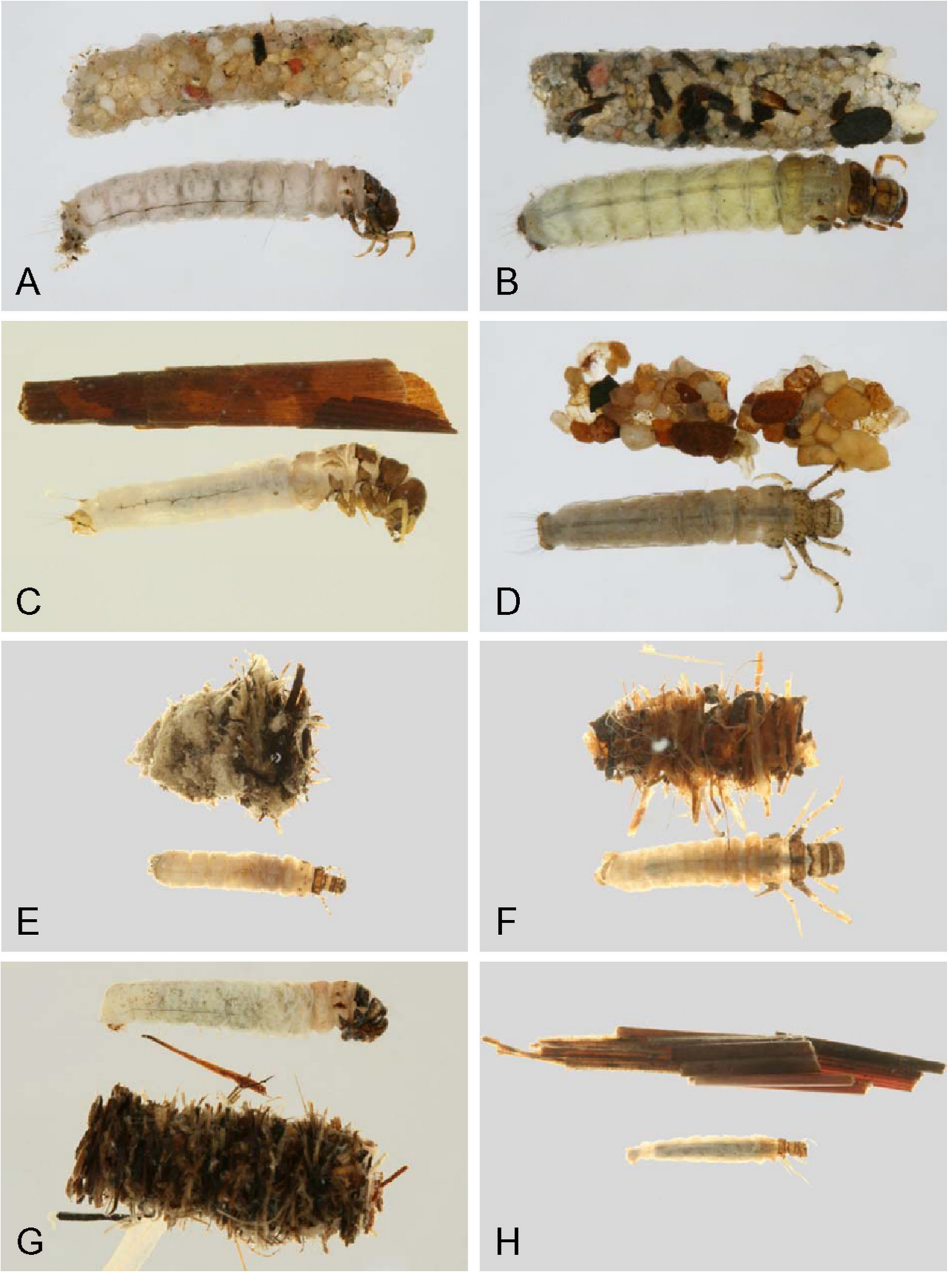
**Limnephilidae A: *Limnephilus ademus, *lateral; B:*Limnephilus alaicus *dorsal; C:*Limnephilus argenteus, *lateral; D:*Limnephilus canadensis *dorsal; E: *Limnephilus externus, *dorsal; F: *Limnephilus extractus*, dorsal; G:*Limnephilus femoralis, *lateral; H:*Limnephilus fischeri *dorsal.**

**Figure 23:**
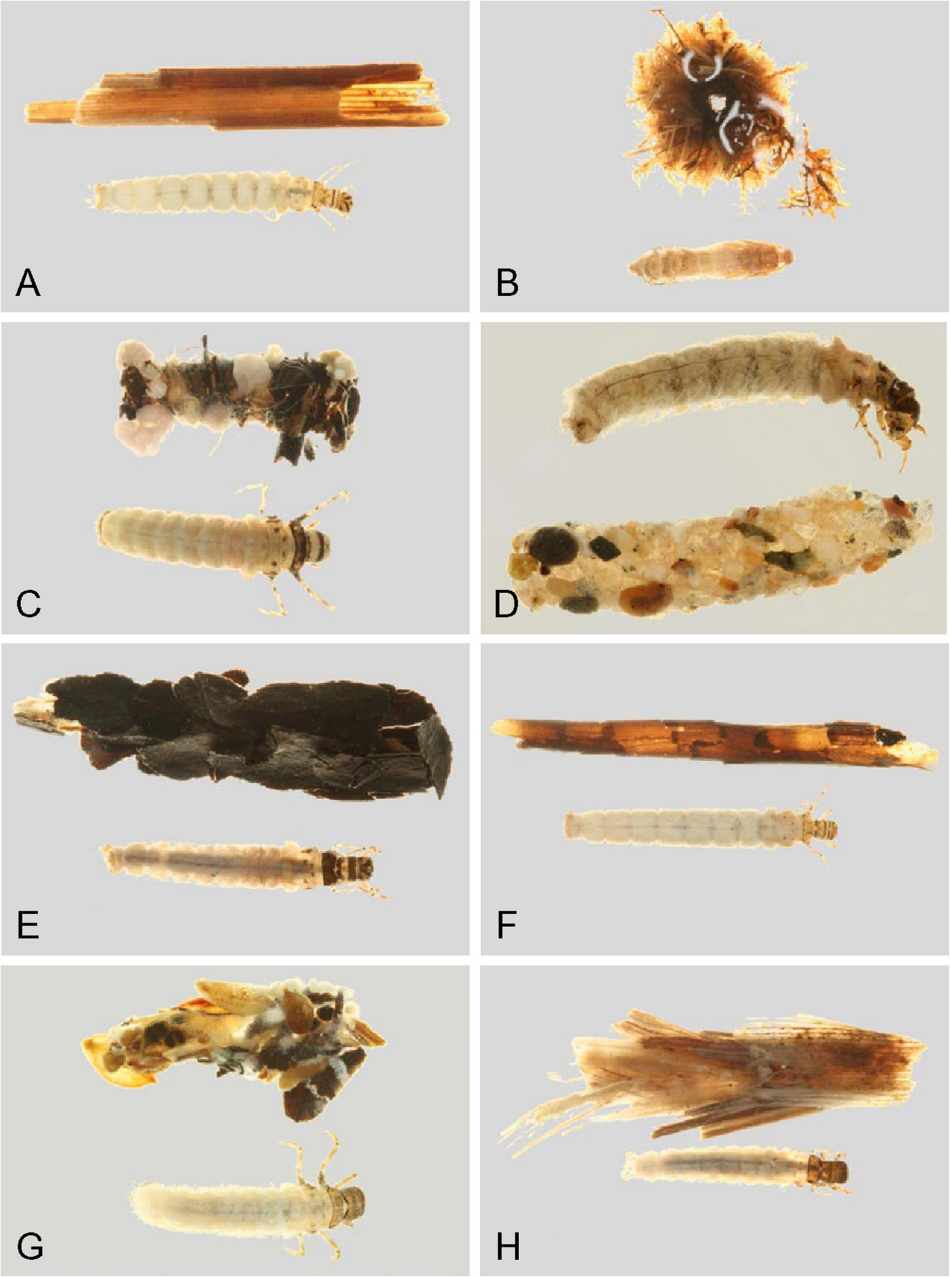
**Limnephilidae A: *Limnephilus hageni, *dorsal; B: *Limnephilus indivisus, *dorsal; C: *Limnephilus infernalis, *dorsal; D: *Limnephilus major, *lateral; E: *Limnephilus nigriceps, *dorsal; F: *Limnephilus partitus, *dorsal; G: *Limnephilus perpusillus, *dorsal; H: *Limnephilus picturatus, *dorsal.**

**Figure 24:**
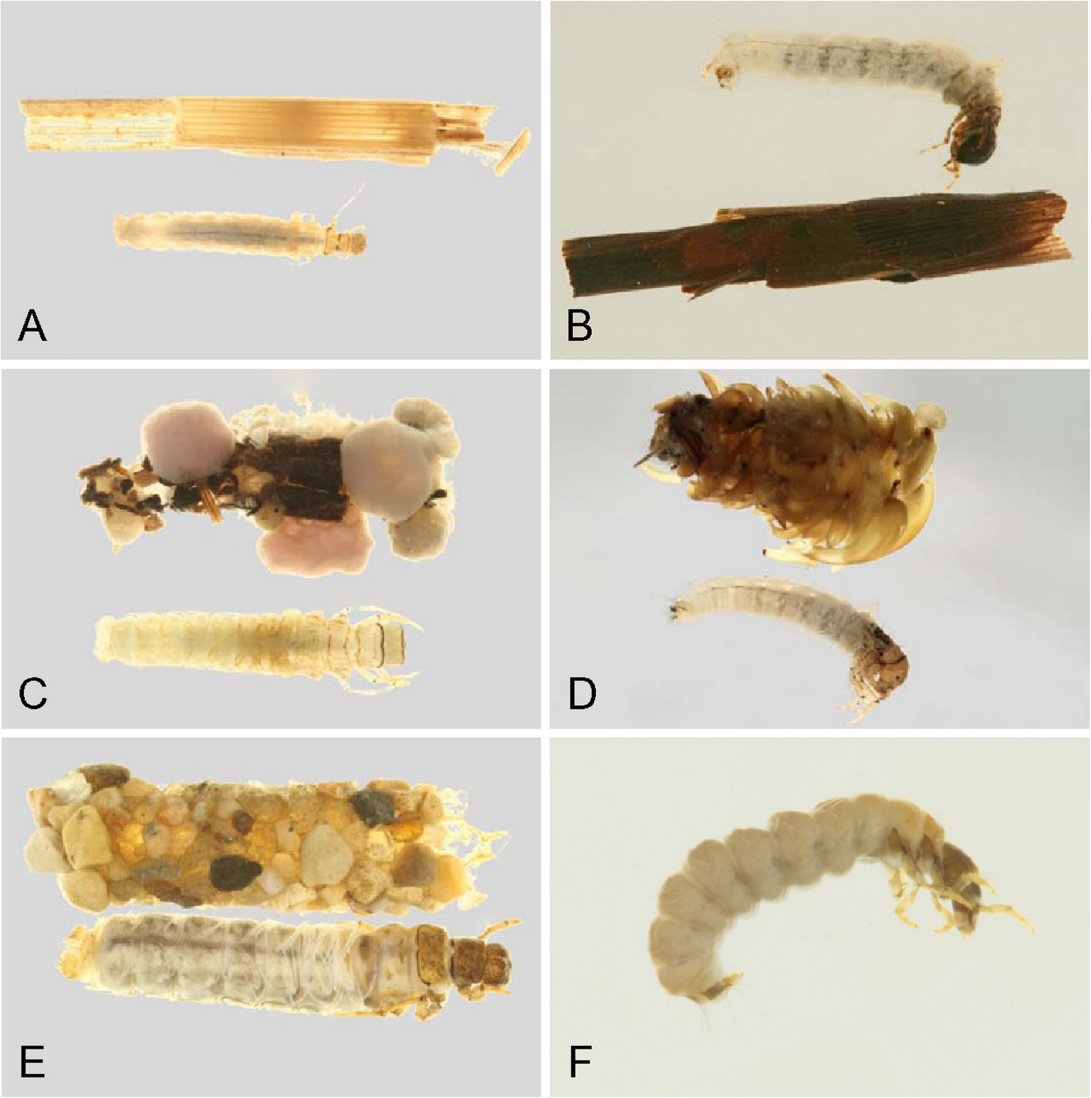
**Limnephilidae A: *Limnephilus sansoni, *dorsal; B: *Limnephilus sericeus, *lateral; C: *Nemotaulius hostilis, *dorsal; D: *Phanocelia canadensis, *lateral; E:*Philarctus bergrothi, *dorsal; Glossosomatidae F: *Glossosoma intermedium, *lateral.**

Through the use of DNA barcoding, this study was able to associate larvae and adults for more than 2/3 of the caddisfly fauna of the Churchill area. An additional 23 species were recorded for the Churchill region since previous reports, increasing the total species count to 91 for the area. Habitus pictures of the collected larvae and cases are contained in Figures 18, 19, 20, 21, 22, 23 and 24. These pictures should not be used for determining genus or species as cases are often not distinct within a species, and in some genera, highly variable. With molecularly identified larvae, unique morphological characters could be quickly distinguished for most larval species, and a taxonomic key for the Limnephilidae was built for the area. Thus, the integration of both DNA barcoding and larval taxonomy has allowed for the rapid identification and description of the Churchill Trichoptera fauna.

Obvious discrepancies in occurrence and abundance between adult or larval stages of many Churchill caddisflies have been observed. For instance, a total of 29 species were only represented by adults, while 11 species only by larvae (Table 1). At least for some species, e.g., *Cheumatopsyche campyla* complex, the collecting of larvae has been mainly limited by our sampling technology (dip net and hand picking) that is not suitable for large and deep river habitats. The failure in collecting either life stages for some species might be also due to their rarity and perhaps also the multi-year life cycle characters. Furthermore, a number of “rare” species defined by availability of adult numbers, have proved to be locally abundant after their larvae were collected, e.g., *Brachycentrus americanus*, *Anabolia bimaculata*, *Limnephilus canadensis*, *L*. *nigriceps, Philarctus bergrothi*. Thus, the sampling of multiple life-stages proved important for conducting comprehensive biodiversity surveys. Of course, the capability of identifying caddisfly larvae from the Churchill area also improves our understanding of the life history and biodiversity distribution (across microhabitats) of these important freshwater macroinvertebrates.

The successful association of a significant portion of Churchill’s caddisfly larvae is also contributing to studies in larval systematics and phylogenetics of several key trichopteran lineages, e.g., Limnephilidae. In Brachycentridae, adults of *Brachycentrus fuliginosus* were collected. This is the only recent record for this rare taxon that we are aware of. Once the larvae are found and associated via DNA, the description of the larvae will complete the larval descriptions for all North American *Brachycentrus*. Another group where the systematics and phylogeny are rapidly changing is Limnephilidae [44]. The many Churchill *Limnephilus* larvae associations will greatly improve our ability to understand the systematics of this group.

While this study demonstrated the successful application of DNA barcoding for linking life stages in Trichoptera, this approach can be applied to other taxonomic groups. Many taxonomic groups also suffer from the same limitations as in Trichoptera, where the taxonomic literature is written for adults and immature rearing is a difficult endeavor. This limitation has been recognized by a number of researchers, who have successfully employed DNA barcoding to associate different life stages of beetles [50], midges [51], earthworms [52], marine planktonic larvae [53], and shrimp [54]. There are also practical applications of this approach, such as the detection of invasive marine diapausing eggs in ship ballast water [55]. Of course, authors have noted [51,56] that the utility of this approach is dependent on a comprehensive reference library of identified adults with COI sequences. However, this issue is less problematic if identifiable adults are simultaneously collected and sequenced in the same local area. In addition, as more COI sequences are generated from identified adults, this issue will become less of an impediment.

While this study has demonstrated the effectiveness of DNA barcoding approaches to understanding biodiversity at a local level and associating different life stages, much more work is required to catalog the North American Trichoptera diversity. It is hoped more taxonomists and field biologists submit material for DNA association with the goal of rapidly improving our ability in North America to determine all life stages to the species level. Funding agencies also need to recognize the value of such analyses and provide concurrent funding for field collection, DNA analysis, and comparative morphological evaluations. Ideally, funding would be provided to examine both adult and larval stages to build upon the current COI reference library, and to contribute a broader understanding of Trichoptera biodiversity and ecology.

The DNA barcode reference library, taxonomic descriptions, and keys generated in this study will be a valuable aid for future studies on Trichoptera larvae in the Churchill region. This work provides researchers the tools to take either a molecular or morphological approach to species-level identification. It is expected that these resources will be of use to future ecological and biodiversity studies in the area.

## Conclusions

Associating different life stages of Trichoptera in the Churchill, MB area by applying both morphology and DNA barcoding proved highly successful. This study has provided researchers with diagnostics for nearly all caddisfly larvae available from the Churchill region to date and a taxonomic key for the limnephilid larvae to utilize in future biodiversity and ecological research in the area.

## Methods

### Specimen collection and sorting

Specimens were collected from the sub-arctic location of Churchill, MB, Canada during July 17 – August 2, 2009, and over a 12-week period from June 5 – August 25, 2010, in addition to prior collecting effort since 2002 [7,8]. Sampling included a variety of freshwater locations including coastal saline and freshwater rock pools, tundra ponds, lakes, creeks, and along the Churchill River. In total, 75 sites were visited once a month for three months in 2010, and these included the sites from which collections were made in 2009.

Trichoptera larvae were collected using a dip net as well as hand collections involving investigating under rocks and debris. Collected specimens were preserved in 95% ethanol and photographed with their case using a Cannon EOS 30D and an EOS 50D. Specimens were identified to family based on Wiggins [37] and further sorted into morphospecies based on variation in head and thoracic markings, case type, habitat sampled, and time collected. For each hypothesized morphospecies, 10 specimens were selected for DNA sequencing except for a few species showing adaptation to a broad range of salinity, where more individuals were analyzed in 2009. If a hypothesized morphospecies contained several lineages based on the COI clustering and barcode identification, further specimens were sampled. All samples are stored at the Biodiversity Institute of Ontario at the University of Guelph, Guelph, ON, Canada.

### Molecular analysis

Specimens had one leg sub-sampled, and molecular methods followed standard manual DNA barcoding protocols [57]. DNA was extracted using an AcroPrep 96 well 3.0 μm glass fibre plate and was eluted with 50 μl of water. Extracted DNA was then amplified for the 658 bp COI region using polymerase chain reaction (PCR) using a 12.5 μl reaction volume. This reaction was comprised of 6.25 μl 10% trehalose (D-(+)-Trehalose dehydrate), 2 μl ddH_2_O, 1.25 μl 10x reaction buffer, 0.625 μl 50 mM MgCl2, 0.0625 μl 10 mM dNTP, 0.06 μl 5 U/μl *Taq* DNA polymerase (Invitrogen), 0.125 μl of 10 μM of both forward and reverse primer, and 2 μl of DNA. The primers used in this study to amplify COI included a primer cocktail of two forward primers: LepF1 - ATTCAACCAATCATAAAGATATTGG, LCO1490 - GGTCAACAAATCATAAAGATATTGG; and two reverse primers: LepR1 - TAAACTTCTGGATGTCCAAAAAATCA, and HCO2198 – TAAACTTCAGGGTGACCAAAAAATCA [58,59], for full-length barcodes. Additionally, two sets of primers (MEPTR1-t1 and MLepR1) paired with routine reverse and forward primers, respectively, targeting the first and second halves of the full-length barcode regions were employed following Zhou et al. [7]. The PCR reaction was thermocycled for 94°C 1 min; 5 cycles of 94°C 40 s, 45°C 40 s, 72°C 1 min; 35 cycles of 94°C 40 s, 51°C 40 s, 72°C 1 min; held at 72°C for 5 min, and stored at 4°C. Successful PCR reactions were checked using an Invitrogen 2% agarose E-gel® with an ethidium bromide stain and developed with UV and if successful, were subsequently bi-directionally sequenced using BigDye® and a Applied Biosystems 3730XL DNA analyzer [60]. All information associated with each specimen, including collection information, taxonomy, photograph, and the COI sequence, were uploaded to BOLD.

### Tree Construction

COI sequences were downloaded from BOLD and combined into unique haplotypes using a script written in Python. All unique COI haplotypes were used to construct a NJ tree using MEGA v5.0 [61], using a Neighbor-Joining method [62] with pairwise deletion of missing sites and Kimura-2-Parameter (K2P) distances [63]. Terminal nodes were collapsed into triangles, where the height represented the number of unique haplotypes and the length represented intraspecific divergence. Species represented by both adult and larval specimens were marked in purple color; those represented only by adults were marked in blue; and those by only larvae were marked by green (Figure 1). Numbers in brackets after each species name represent the number of unique COI haplotypes and the number of individuals sequenced, respectively.

### Larval-Adult Association

This work has used COI to verify larval associations, a process which can greatly reduce the time and effort necessary to associate larvae with the corresponding adult, especially when metamorphotypes [64] cannot be located at the time of collection. The molecular identification of larval specimens followed criteria proposed by Zhou et al. [5]. Briefly, a species was assigned to a larval specimen when its DNA barcode shared identical sequence with a barcode reference obtained from an identifiable adult specimen (typically a male specimen), or alternatively, if the larval sequence fell in a species boundary defined by adult sequences on a phylogenetic tree.

### Morphology

Morphological comparisons were made for all DNA determined larval taxa. At least two specimens of each taxon were compared for variability whenever possible. When only limited/immature specimens were available for morphological and DNA character analysis, it is noted in the text. Illustrations were prepared and processed for characteristic structures with the use of compound and stereo microscopes, Xnview©, Automontage©, Zerene Stacker©, and Photoshop©. Material extraneous to the character in question was often removed electronically and other characters such as a characteristic seta were occasionally added when the original structure was broken. Terminology follows that of Wiggins [21].

The larval keys presume the readers have suitable publications for determining specimens to genus [21,64]. Habitus pictures of the available larvae and cases are contained in Figures 18, 19, 20, 21, 22, 23 and 24. These pictures should not be used for determining genus or species as cases are often not distinct within a species, and in some genera, highly variable. We have selected habitus pictures based on their availability and clarity.

## Competing interests

The authors declare that they have no competing interests.

## Authors’ contributions

All authors were involved in the conceptual design. EEB and XZ carried out the collection and sorting of specimens. DER and XZ performed the morphological identifications. DER wrote the species diagnoses, taxonomic description, and key. DER, EEB, and the Imaging Department at the Biodiversity Institute of Ontario took the photographs. EEB and XZ performed the DNA sequencing, editing, and alignment. XZ performed the molecular analysis. All authors helped draft the text and approved the final manuscript.
